# Antithrombotic Agents for tPA‐Induced Cerebral Hemorrhage: A Systematic Review and Meta‐Analysis of Preclinical Studies

**DOI:** 10.1161/JAHA.120.017876

**Published:** 2020-12-05

**Authors:** Yang Ye, Fu‐Tao Zhang, Xiao‐Yi Wang, Hong‐Xuan Tong, Yu‐Tian Zhu

**Affiliations:** ^1^ Department of Integration of Chinese and Western Medicine School of Basic Medical Sciences Peking University Beijing China; ^2^ Tasly Microcirculation Research Center Peking University Health Science Center Beijing China; ^3^ University of Chinese Academy of Sciences Beijing China; ^4^ Northeast Institute of Geography and Agroecology Chinese Academy of Sciences Harbin China; ^5^ National Engineering Laboratory for Improving Quality of Arable Land Institute of Agricultural Resources and Regional Planning Chinese Academy of Agricultural Sciences Beijing China; ^6^ Institute of Basic Theory for Chinese Medicine China Academy of Chinese Medical Sciences Beijing China; ^7^ Department of Urology Peking University Third Hospital Beijing China

**Keywords:** animal model, antithrombotic, hemorrhagic transformation, ischemic stroke, tPA (tissue‐type plasminogen activator), Ischemic Stroke, Basic Science Research, Anticoagulants, Thrombosis

## Abstract

**Background:**

tPA (tissue‐type plasminogen activator) remains the only approved drug for acute ischemic stroke, with a potentially serious adverse effect: hemorrhagic transformation. The effects of antithrombotic agents on tPA‐induced hemorrhagic transformation after ischemic stroke are not clearly defined. We performed a systematic review and meta‐analysis in preclinical studies aiming to evaluate the efficacy of antithrombotic agents on tPA‐induced hemorrhagic transformation after ischemic stroke.

**Methods and Results:**

We conducted a systematic review and meta‐analysis of studies testing antithrombotic agents in animal models of tPA‐induced hemorrhagic transformation. The pooled effects were calculated using random‐effects models, and heterogeneity was explored through meta‐regression and subgroup analyses. Publication bias was assessed using trim and fill method and the Egger test. The efficacy of 18 distinct interventions was described in 22 publications. The pooled data showed a significant improvement in cerebral hemorrhage, infarct size, and neurobehavioral outcome in treated compared with control animals (standardized mean difference, 0.45 [95% CI, 0.11–0.78]; standardized mean difference, 1.18 [95% CI, 0.73–1.64]; and standardized mean difference, 0.91 [95% CI, 0.49–1.32], respectively). Subgroup analysis indicated that quality score, random allocation, control of temperature, anesthetic used, stroke model used, route of drug delivery, time of drug administration, and time of assessment were significant factors that influenced the effects of interventions.

**Conclusions:**

Administration with antiplatelet agents revealed statistically significant improvement in all the outcomes. Anticoagulant agents showed significant effects in infarct size and neurobehavioral score, but fibrinolytic agents did not show any significant improvement in all the outcomes. The conclusions should be interpreted cautiously given the heterogeneity and publication bias identified in this analysis.

**Nonstandard Abbreviations and Acronyms**
HT: hemorrhagic transformation
RESTART: Restart or Stop Antithrombotics Randomized Trial
RETRACE: German‐Wide Multicenter Analysis of Oral Anticoagulation Associated Intracerebral Hemorrhage
SMD: standardized mean difference

**Clinical Perspective**

**What Is New?**

- Antithrombotic therapy resulted in significant improvement of cerebral hemorrhage, infarct size, and neurobehavioral score in animal models of tPA (tissue‐type plasminogen activator)–induced hemorrhagic transformation.
- Study quality score, random allocation, control of temperature, anesthetic used, stroke model, route of drug delivery, time of drug administration, and time of assessment were significant factors that influenced the effects of antithrombotic agents.

**What Are the Clinical Implications?**

- Our results revealed that antiplatelet agents and anticoagulant agents were the more effective subtypes of antithrombotic agents, whereas fibrinolytic agents produced no significant differences.
- Results of this analysis provided guidance for future animal studies investigating the efficacy of antithrombotic agents on tPA‐induced hemorrhagic transformation and valuable insight into clinical drug use and trial design.

Early intravenous thrombolysis with tPA (tissue‐type plasminogen activator) remains the only US Food and Drug Administration approved agent for acute ischemic stroke treatment. However, tPA can lead to an increased risk of hemorrhagic transformation (HT), especially beyond 4.5 hours of stroke onset.^1^ HT is often associated with significant mortality and disability rate.^2^ Therefore, it is important to control the risk of HT when tPA is used in patients after acute ischemic stroke.

Antithrombotic agents are widely used to prevent and treat thrombosis in the venous and arterial circulations.^3^ After acute ischemic stroke, antithrombotic therapy is recommended to prevent recurrent thromboembolism.^4^ However, when antithrombotic drugs are administrated after ischemic stroke, the risk of thromboembolic events may decrease, whereas the risk for bleeding increases.^5^ The use of antithrombotic agent should be tailored to maintain a balance between preventing thromboembolic events and limiting bleeding risks. Given that tPA itself may lead to an increased risk of HT, there is controversy about the safety of antithrombotic therapy after tPA thrombolysis‐induced intracerebral hemorrhage. Moreover, antithrombotic agents can be divided into antiplatelets, anticoagulants, and fibrinolytics, based on the mechanism of action.^6^ The safety of different classes of antithrombotic drugs after tPA‐induced intracerebral hemorrhage also remains controversial.

Herein, we reported a systematic review and meta‐analysis of data from preclinical studies testing the efficacy of antithrombotic agents on tPA‐induced HT. The purpose of this study was to determine the effects of antithrombotic agents on cerebral hemorrhage, infarct size, and neurobehavioral score in animal models of tPA‐induced HT. We also identified the factors that influenced the efficacy of antithrombotic agents. Results of this review will guide future animal studies that seek to elucidate the efficacy of antithrombotic agents on tPA‐induced HT, as well as provide valuable insight into clinical drug use and trial design.

## Methods

The data that support the findings of this study are available from the corresponding author on reasonable request. We applied the Collaborative Approach to Meta‐Analysis and Review of Animal Data From Experimental Stroke checklist for systematic reviews of animal models in stroke.^7^ We also followed the Preferred Reporting Items for Systematic Reviews and Meta‐Analyses guidelines to perform the systematic review and meta‐analysis. The primary outcome measure was cerebral hemorrhage. The secondary outcome measures were infarction size and neurobehavioral score.

### Search Strategy

Animal studies assessing the effects of antithrombotic agents in models of tPA‐induced HT after stroke were identified from the following electronic databases: PubMed, Web of Science, and Scopus. No date limit was placed on the search. The search was performed on February 12, 2020, and updated on April 20, 2020. The search strategy is shown in detail in Data S1. Searches were limited to English‐language publications.

### Inclusion and Exclusion Criteria

We included studies that reported the efficacy of antithrombotic agents for tPA‐induced HT in animal models of stroke. Studies that described the effects of interventions compared with a control group receiving vehicle or no treatment in animal models of tPA‐induced HT were eligible for inclusion. We included studies that quantified cerebral hemorrhage as an outcome (hemoglobin content and hemorrhage volume/area/score) (Table S1). Hemoglobin content was chosen as preferred indicator in these measurement indicators because of its precision. Any animal species and any route of delivery of intervention at any time point were included. We excluded studies that did not report the number of animals per group. Clinical studies, review articles, abstract‐only publications, and non–English‐language publications were excluded. Studies were not included if the control groups were not appropriate. Studies that only quantified the incidence of HT were also excluded.

### Data Extraction

We extracted data from the included studies on the publication details (author and year), animal used (sex, species, and strain), type of stroke model, intervention used (route, dose, and timing), anesthetic used, tPA administration (dose and timing), and details of the outcome measures (hemorrhage, infarction, and neurobehavioral outcome). For each comparison, we extracted data reporting the sample size per group, mean value, and variance (SD or SEM) for both the control and treatment group. Study factors were examined one at a time without adjustment for other factors.

When multiple treatment groups shared a single control group, the sample size of this control group was divided by the number of treatment groups to adjust the impact of this control group.^8^ When treatment was administered in multiple doses, only the dose with the best efficacy in treatment group was included. When outcomes were assessed for >1 time point, only the last time of assessment was extracted.^9^ When we were unsure of whether the measure of variance reported was SD or SEM, the measure was assumed as SEM to insure a study might not be given undue weight in the meta‐analysis.^8^ When data were only presented graphically, the ImageJ software (National Institutes of Health) was used to quantify the results. When data required were missing, we contacted authors to request the data. When the essential data could not be obtained, the studies will be excluded from further analysis.

### Quality Assessment

We assessed risk of bias using a 10‐item checklist of Collaborative Approach to Meta‐Analysis and Review of Animal Data From Experimental Stroke,^7^ comprising the following: (1) publication in a peer‐reviewed journal, (2) control of temperature, (3) random allocation to groups, (4) allocation concealment (blinded induction of ischemia), (5) blinded assessment of outcome, (6) use of an anesthetic without intrinsic neuroprotective activity (ketamine), (7) the use of comorbid animals, (8) performing a sample size calculation, (9) compliance with animal welfare regulations, and (10) statement of potential conflicts of interest.

### Statistical Analysis

For cerebral hemorrhage, infarct size, and neurobehavioral outcome, a standardized mean difference (SMD) effect size was used to standardize the results to a uniform scale.

The SMD values were calculated using Review Manager software and pooled in a weighted mean difference meta‐analysis using a random‐effects model.^10^ When the pooled SMD effect size was >0, it can be defined as an improvement. The improvement was statistically significant when the 95% CI of SMD was >0. When one outcome from one cohort of animals was evaluated by different measures at the same time point, the results were combined in random‐effects meta‐analyses. Heterogeneity was assessed using the Q statistic and quantified using the I^2^ statistic.^11^ The source of heterogeneity was explored by meta‐regression and subgroup analysis. Meta‐regressions were conducted to evaluate the impact of components of the study quality checklist and study characteristics. Subgroup analyses were conducted to explore the impacts of interventions with different mechanisms and characteristics. Publication bias was assessed using funnel plots, trim and fill method,^12^ and the Egger test.^13^ Sensitivity analysis was also performed to confirm the stability of the results. Statistical analyses were performed using Review Manager (version 5.3) and STATA (version 13.1) software.

## Results

### Study Characteristics

**Figure 1:**
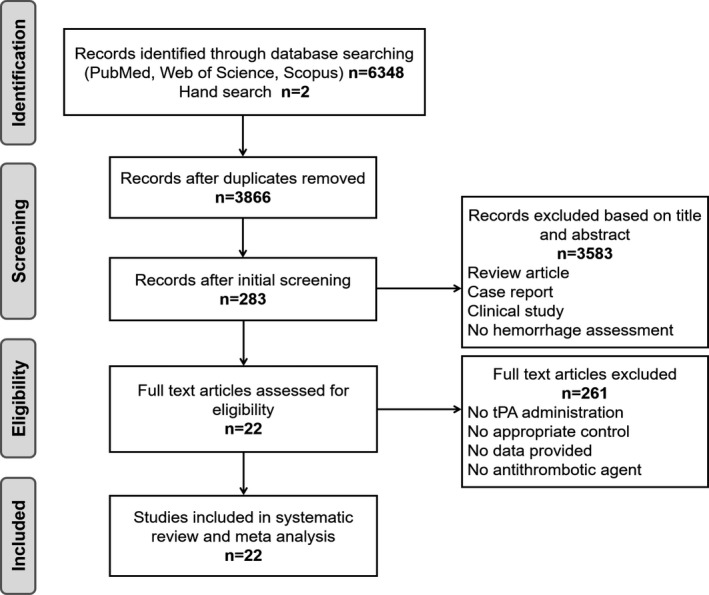
Flow diagram of publication inclusion. tPA indicates tissue‐type plasminogen activator.

Our final search was performed on April 20, 2020. We identified 1866 articles on PubMed, 3857 articles on Web of Science, 625 articles on Scopus, and 2 articles by hand search. After excluding 6328 publications, 22 articles were finally included in our analysis (Figure 1). The 22 articles contained 44 comparisons for the primary outcome, 35 comparisons for infarct size, and 29 comparisons for neurobehavioral outcome. Eighteen different antithrombotic agents were used in the included studies, and they were categorized into 3 subtypes: antiplatelet agents, anticoagulant agents, and fibrinolytic agents. All included studies and study characteristics are listed in the Table.^14^, ^15^, ^16^, ^17^, ^18^, ^19^, ^20^, ^21^, ^22^, ^23^, ^24^, ^25^, ^26^, ^27^, ^28^, ^29^, ^30^, ^31^, ^32^, ^33^, ^34^, ^35^

### Study Quality

**Figure 2:**
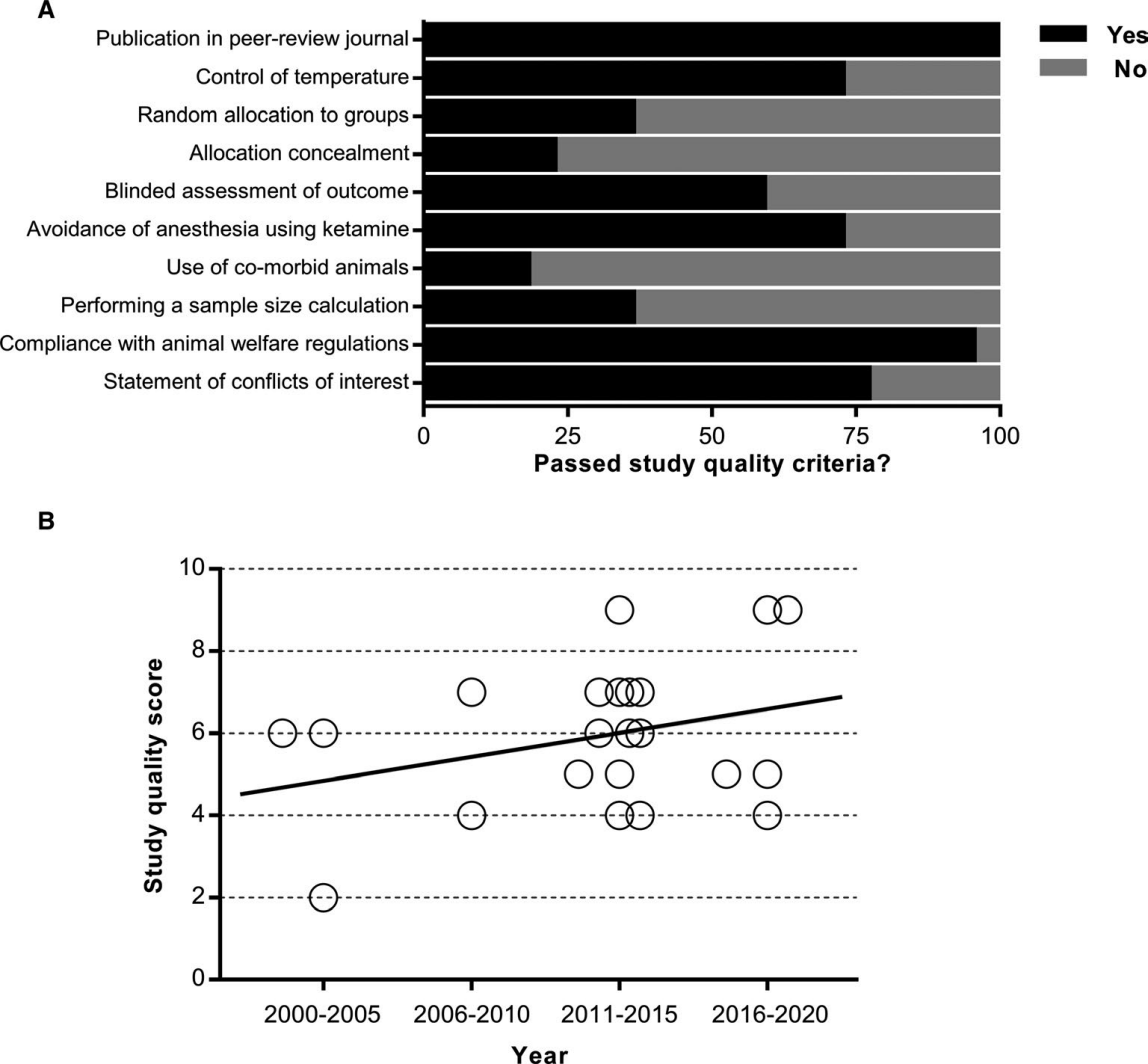
Study quality assessment of the included publications. Study quality was assessed using a 10‐item checklist of Collaborative Approach to Meta‐Analysis and Review of Animal Data From Experimental Stroke on reporting in publications (**A**). Values are expressed as the percentage of studies reporting each quality indicator. The correlation between study quality and year of publication (**B**).

The median study quality score was 6 of a possible 10 (interquartile range, 5–7). All publications included in this analysis were published in peer‐reviewed journals (Figure 2A). Sixteen publications (73%) reported control of temperature during surgery. Eight publications (36%) reported random allocation to treatment groups and control groups. Only 5 publications (23%) reported blinded induction of stroke. Blinded assessment of outcome was reported in 13 publications (59%), and anesthesia without using ketamine was reported in 16 publications (73%). Three publications used hypertensive animals, 1 publication used hyperglycemic animals, and the remaining 18 publications (82%) did not model a relevant comorbidity. Eight publications (36%) reported having performed a sample size calculation. Only 1 publication (5%) did not reported compliance with animal welfare regulations. Seventeen publication (77%) reported whether a conflict of interest existed. There was a statistically significant correlation between study quality and year of publication, with newer studies giving higher quality scores (*R* ^2^=96.16%; *P*=0.0194; Figure 2B). The reported study quality score for each article included in this review is summarized in Table S2.

### Meta‐Analysis

**Figure 3:**
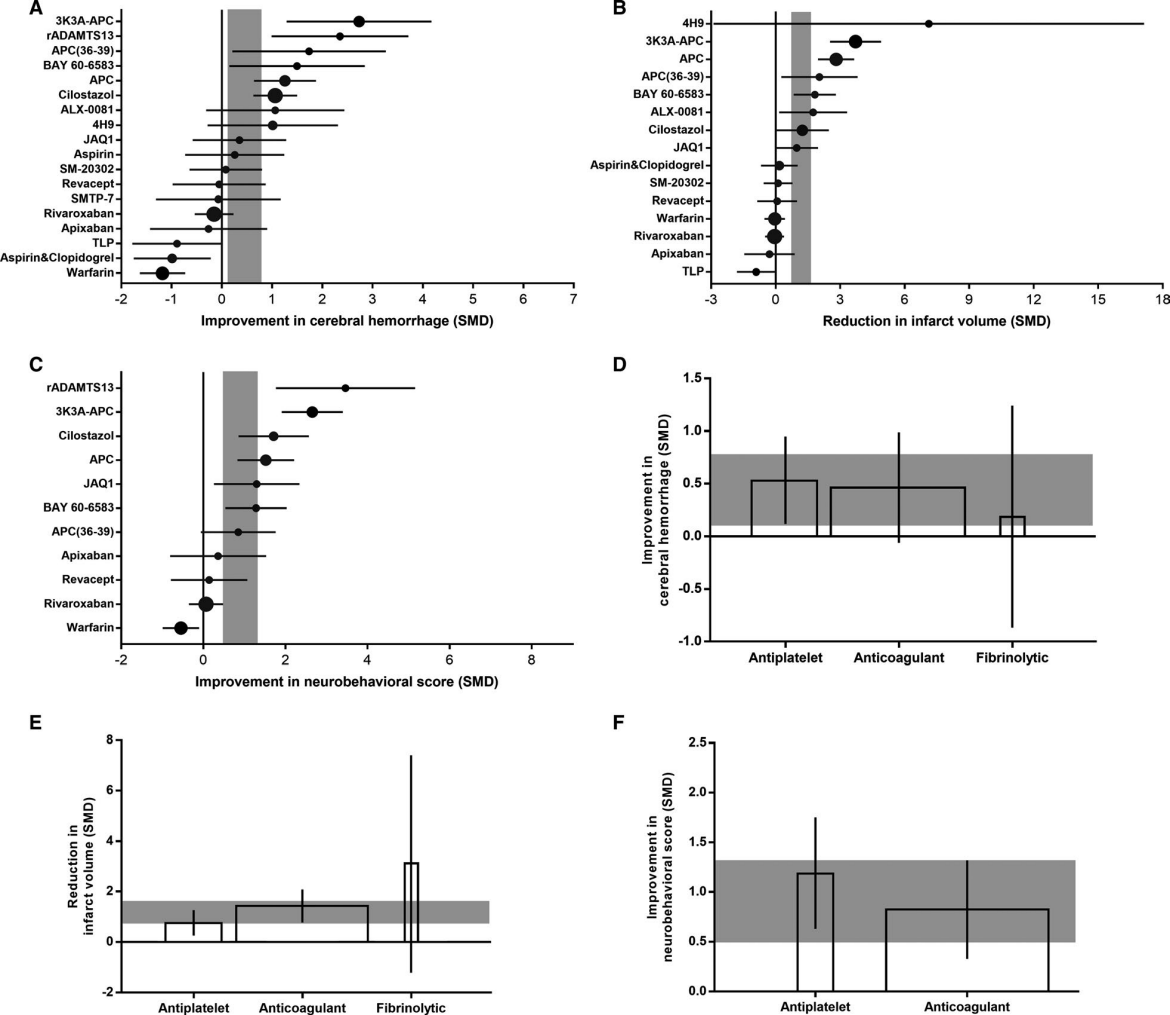
Efficacy of antithrombotic agents on cerebral hemorrhage, infarct size, and neurobehavioral score. Timber plots of the effect size in cerebral hemorrhage (**A**), infarct size (**B**), and neurobehavioral score (**C**), calculated using standardized mean differences (SMDs). Symbol sizes represent the relative number of animals tested for each intervention. The horizontal error bars represent the 95% CI of individual studies. The vertical gray bars represent the 95% CI of the pooled estimate of efficacy. Efficacy of antithrombotic subtypes (antiplatelet agents, anticoagulant agents, and fibrinolytic agents) on cerebral hemorrhage (**D**), infarct size (**E**), and neurobehavioral score (**F**). The width of each bar represents the relative number of animals in that subgroup. The vertical error bars represent the 95% CI for the individual estimates, and the horizontal gray bars represent the 95% CI of the pooled estimate of efficacy. APC, activated protein C; APC(36‐39), activated protein C (36‐39); 3K3A‐APC, 3K3A‐activated protein C; rADAMTS13, recombinant ADAMTS13; SMTP‐7, stachybotrys microspora triprenyl phenol‐7; TLP, thrombolysis products.

Overall, cerebral hemorrhage was improved by an SMD of 0.45 (95% CI, 0.11–0.78) in experiments testing 18 antithrombotic agents, with substantial heterogeneity between studies (χ^2^=155.28; I^2^=72%; *df*=43; *P*<0.001; Figure 3A and Figure S1). Infarct size was reduced by an SMD of 1.18 (95% CI, 0.73–1.64) in experiments testing 15 antithrombotic agents, with substantial heterogeneity between studies (χ^2^=151.02; I^2^=77%; *df*=34; *P*<0.001; Figure 3B and Figure S2). Neurobehavioral score was improved by an SMD of 0.91 (95% CI, 0.49–1.32) in experiments testing 11 antithrombotic agents, with substantial heterogeneity between studies (χ^2^=104.61; I^2^=73%; *df*=28; *P*<0.001; Figure 3C and Figure S3).

### Meta‐Regression and Subgroup Analyses

The pooled estimates for included studies in all meta‐analyses exhibited substantial heterogeneity. Meta‐regression and subgroup analyses were conducted to identify the source of heterogeneity (drug subtype, quality score, random allocation, blinded assessment of outcome, sample size estimate, control of temperature, anesthetic used, stroke model, animal species, route of drug delivery, time of drug administration, and time of assessment).

The antithrombotic agents were categorized into 3 subtypes on the basis of differential mechanisms of action: antiplatelet agents, anticoagulant agents, and fibrinolytic agents (Table). Analysis showed drug subtype did not account for the between‐study heterogeneity or significantly contribute to different estimates of efficacy for cerebral hemorrhage (adjusted *R* ^2^=−6.68%; *P*=0.83; Figure 3D), infarct size (adjusted *R* ^2^=−11.12%; *P*=0.19; Figure 3E), and neurobehavioral score (adjusted *R* ^2^=−0.62%; *P*=0.34; Figure 3F). However, there were still some differences between different subtypes. Pooled data indicated antiplatelet agents were the only type of interventions that improved the cerebral hemorrhage significantly (SMD, 0.53; 95% CI, 0.12–0.95; Figure 3D). For infarct volume and neurobehavioral score, antiplatelet agents (SMD, 0.77 [95% CI, 0.26–1.27]; and SMD, 1.19 [95% CI, 0.63–1.75], respectively) and anticoagulant agents (SMD, 1.43 [95% CI, 0.78–2.08]; and SMD, 0.82 [95% CI, 0.33–1.32], respectively) both showed significant improvement (Figure 3E and 3F). However, fibrinolytic agents did not contribute significantly to the beneficial effects of antithrombotic agents (Figure 3D and 3E).

**Figure 4:**
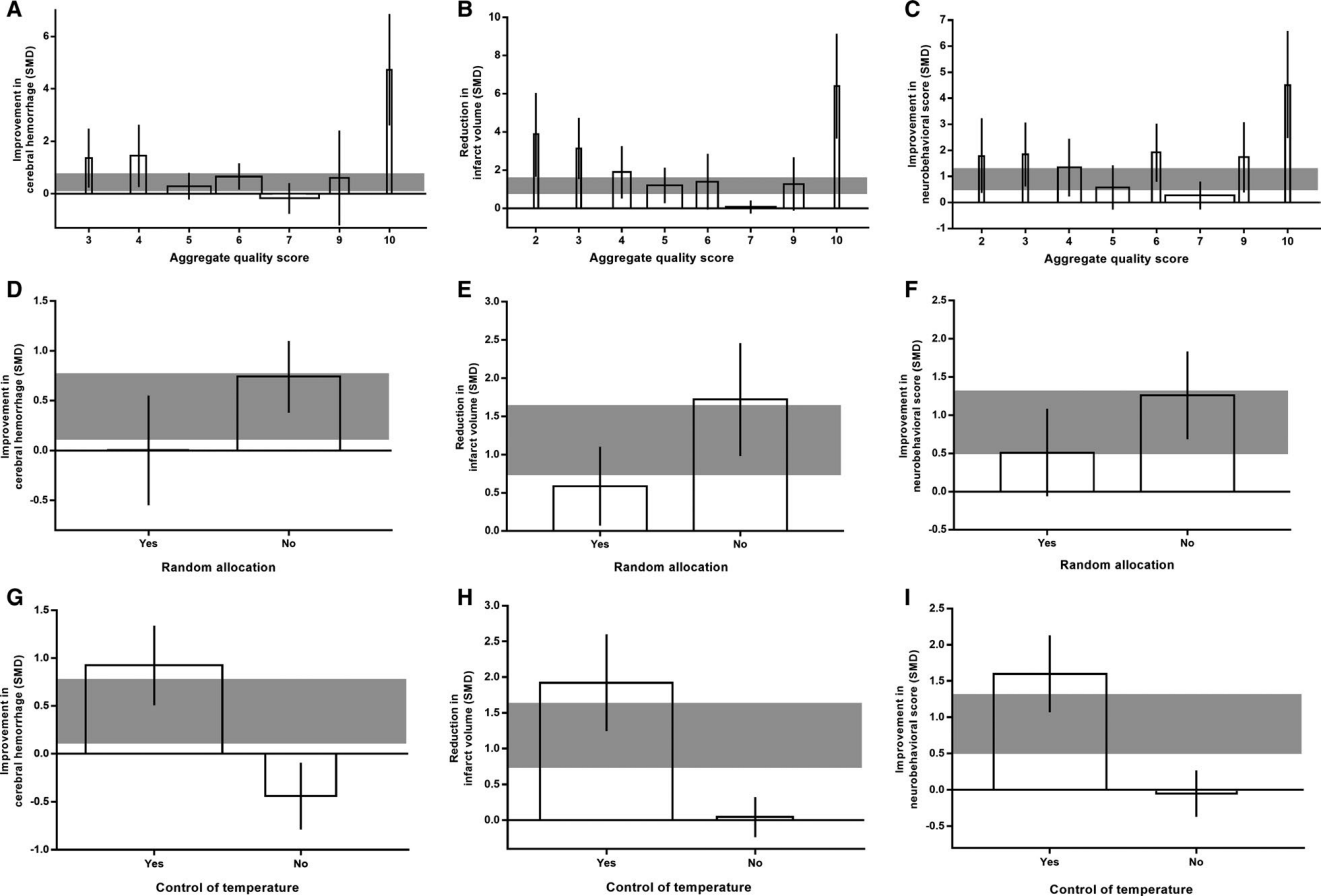
Effect of study quality, random allocation, and body temperature control during surgery on the improvement of antithrombotic therapy. Effect of study quality on cerebral hemorrhage (**A**), infarct size (**B**), and neurobehavioral score (**C**). Effect of random allocation on cerebral hemorrhage (**D**), infarct size (**E**), and neurobehavioral score (**F**). Effect of body temperature control during surgery on cerebral hemorrhage (**G**), infarct size (**H**), and neurobehavioral score (**I**). The width of each bar represents the relative number of animals in that subgroup. The vertical error bars represent the 95% CI for the individual estimates, and the horizontal gray bars represent the 95% CI of the pooled estimate of efficacy. SMD indicates standardized mean difference.

**Figure 5:**
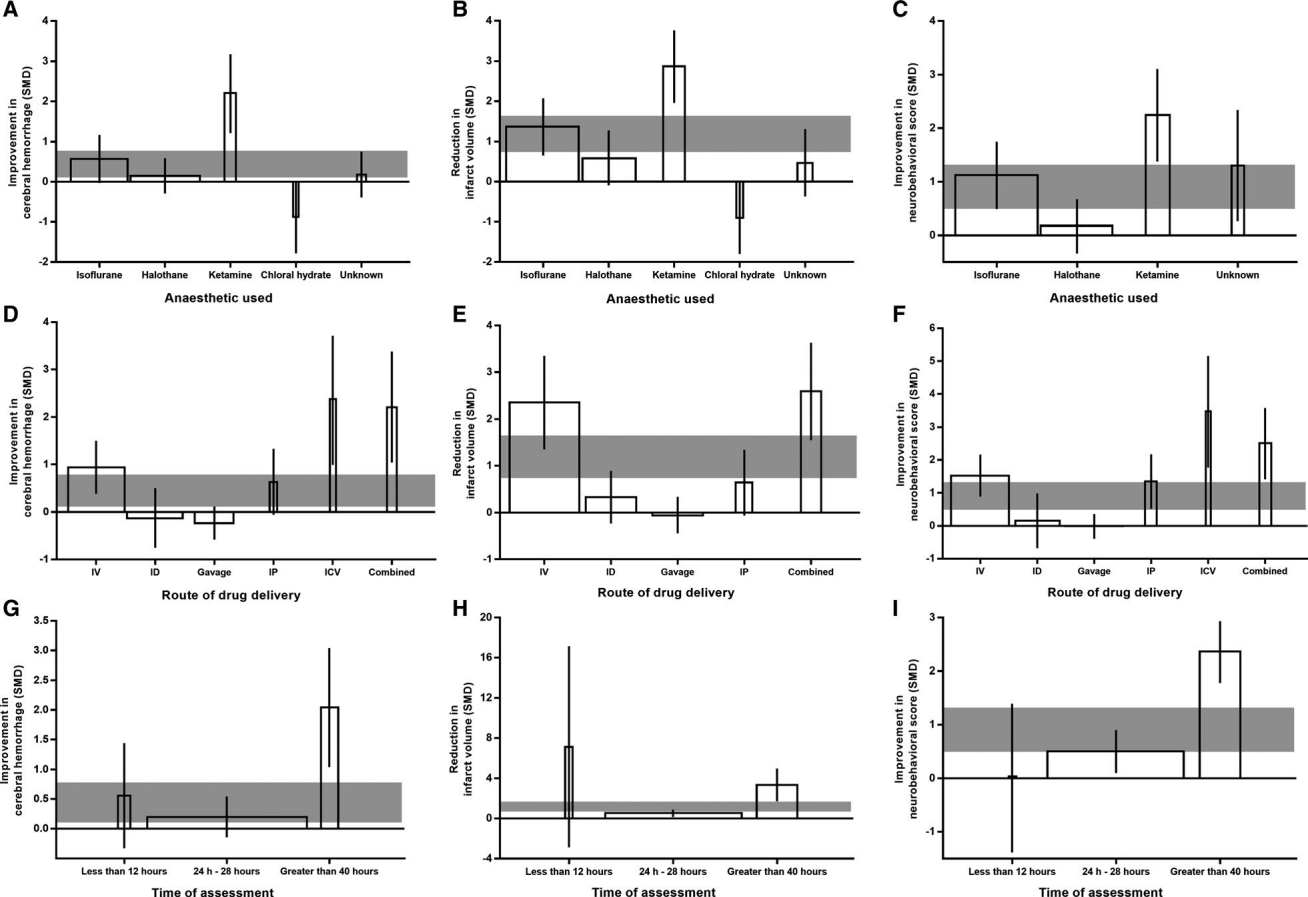
Effect of anesthetic used, route of drug delivery, and time of outcome assessment on the improvement of antithrombotic therapy. Effect of anesthetic used on cerebral hemorrhage (**A**), infarct size (**B**), and neurobehavioral score (**C**). Effect of route of drug delivery on cerebral hemorrhage (**D**), infarct size (**E**), and neurobehavioral score (**F**). Effect of outcome assessment time on cerebral hemorrhage (**G**), infarct size (**H**), and neurobehavioral score (**I**). The width of each bar represents the relative number of animals in that subgroup. The vertical error bars represent the 95% CI for the individual estimates, and the horizontal gray bars represent the 95% CI of the pooled estimate of efficacy. ICV indicates intracerebroventricular; ID, in diet; IP, intraperitoneal; IV, intravenous; and SMD, standardized mean difference.

For cerebral hemorrhage, treatment effects were lower in studies that received a higher aggregate quality score (adjusted *R* ^2^=−0.14%; *P*<0.001; Figure 4A) and in studies that reported random allocation of animals (adjusted *R* ^2^=18.62%; *P*=0.03; Figure 4D). Studies that did not report blinded assessment of outcome reported larger effect size (adjusted *R* ^2^=17.3%; *P*=0.01; Figure S4A). Efficacy was lower in experiments where sample size estimate had been reported (adjusted *R* ^2^=23.93%; *P*=0.01; Figure S5A). Larger effects were seen in studies that reported control of animal body temperature during the induction of stroke (adjusted *R* ^2^=39.62%; *P*<0.001; Figure 4G). The anesthetic used accounted for 15.56% (adjusted *R* ^2^; *P*<0.001; Figure 5A) of the observed heterogeneity. Treatment effects were significantly higher in studies that reported use of anesthetics with ketamine (SMD, 2.19; 95% CI, 1.21–3.18) and lower in studies that reported use of anesthetics with chloral hydrate (SMD, −0.89; 95% CI, −1.78 to 0). We found no significant impact of the stroke model (adjusted *R* ^2^=1.56%; *P*=0.16; Figure S6A) and species (adjusted *R* ^2^=−12.4%; *P*=0.63; Figure S7A). Route of drug delivery (adjusted *R* ^2^=38%; *P*<0.001; Figure 5D), time of drug administration (adjusted *R* ^2^=30.15%; *P*<0.001; Figure S8A), and time of assessment (adjusted *R* ^2^=10.26%; *P*=0.003; Figure 5G) accounted for a significant proportion of the between‐study heterogeneity.

For infarct size, treatment effects were also lower in studies that received a higher aggregate quality score (adjusted *R* ^2^=2.37%; *P*<0.001; Figure 4B) and in studies that reported random allocation of animals (adjusted *R* ^2^=10.52%; *P*=0.01; Figure 4E). Studies that reported blinded assessment of outcome did not show lower effect size (adjusted *R* ^2^=−6.3%; *P*=0.66; Figure S4B). Efficacy tended to be lower in experiments where sample size estimate had been reported (adjusted *R* ^2^=5.33%; *P*=0.1; Figure S5B). Larger effects were seen in studies that reported control of animal body temperature during the induction of stroke model (adjusted *R* ^2^=34.75%; *P*<0.001; Figure 4H). The anesthetic used accounted for 28.28% (adjusted *R* ^2^=27.54%; *P*<0.001; Figure 5B) of the observed heterogeneity. Treatment effects were significantly higher in studies that reported use of anesthetics with ketamine (SMD, 2.86; 95% CI, 1.96–3.76) and lower in studies that reported use of anesthetics with chloral hydrate (SMD, −0.91; 95% CI, −1.8 to −0.02). Studies that used embolic stroke model reported higher effects compared with transient middle cerebral artery occlusion model (adjusted *R* ^2^=43.79%; *P*<0.001; Figure S6B). Species was also found to be associated with treatment effects (adjusted *R* ^2^=−12.98%; *P*=0.03; Figure S7B). Route of drug delivery (adjusted *R* ^2^=37.84%; *P*<0.001; Figure 5E), time of drug administration (adjusted *R* ^2^=37.12%; *P*<0.001; Figure S8B), and time of assessment (adjusted *R* ^2^=36.2%; *P*=0.002; Figure 5H) accounted for a significant proportion of the between‐study heterogeneity.

For neurobehavioral outcome, studies that received a lower aggregate quality score (adjusted *R* ^2^=−5.87%; *P*<0.001; Figure 4C) and did not report random allocation of animals (adjusted *R* ^2^=10.88%; *P*=0.07; Figure 4F) tended to have higher treatment effects. However, studies that reported blinded assessment of outcome gave inflated estimates of efficacy (adjusted *R* ^2^=10.67%; *P*=0.008; Figure S4C). Efficacy tended to be lower in experiments where sample size estimate had been reported (adjusted *R* ^2^=11.86%; *P*=0.09; Figure S5C). Significantly larger effects were seen in studies that reported control of animal body temperature during surgery (adjusted *R* ^2^=60.18%; *P*<0.001; Figure 4I). The anesthetic used accounted for 28.81% (adjusted *R* ^2^; *P*<0.001; Figure 5C) of the observed heterogeneity. Treatment effects were significantly higher in studies that reported use of anesthetics with ketamine (SMD, 2.24; 95% CI, 1.38–3.11). Studies that used embolic stroke model reported higher effects compared with transient middle cerebral artery occlusion model (adjusted *R* ^2^=40.61%; *P*<0.001; Figure S6C). We found no significant impact of the species (adjusted *R* ^2^=−5.77%; *P*=0.82; Figure S7C). Route of drug delivery (adjusted *R* ^2^=76.51%; *P*<0.001; Figure 5F), time of drug administration (adjusted *R* ^2^=72.79%; *P*<0.001; Figure S8C), and time of assessment (adjusted *R* ^2^=35.71%; *P*<0.001; Figure 5I) accounted for a significant proportion of the between‐study heterogeneity.

### Publication Bias and Sensitivity Analysis

**Figure 6:**
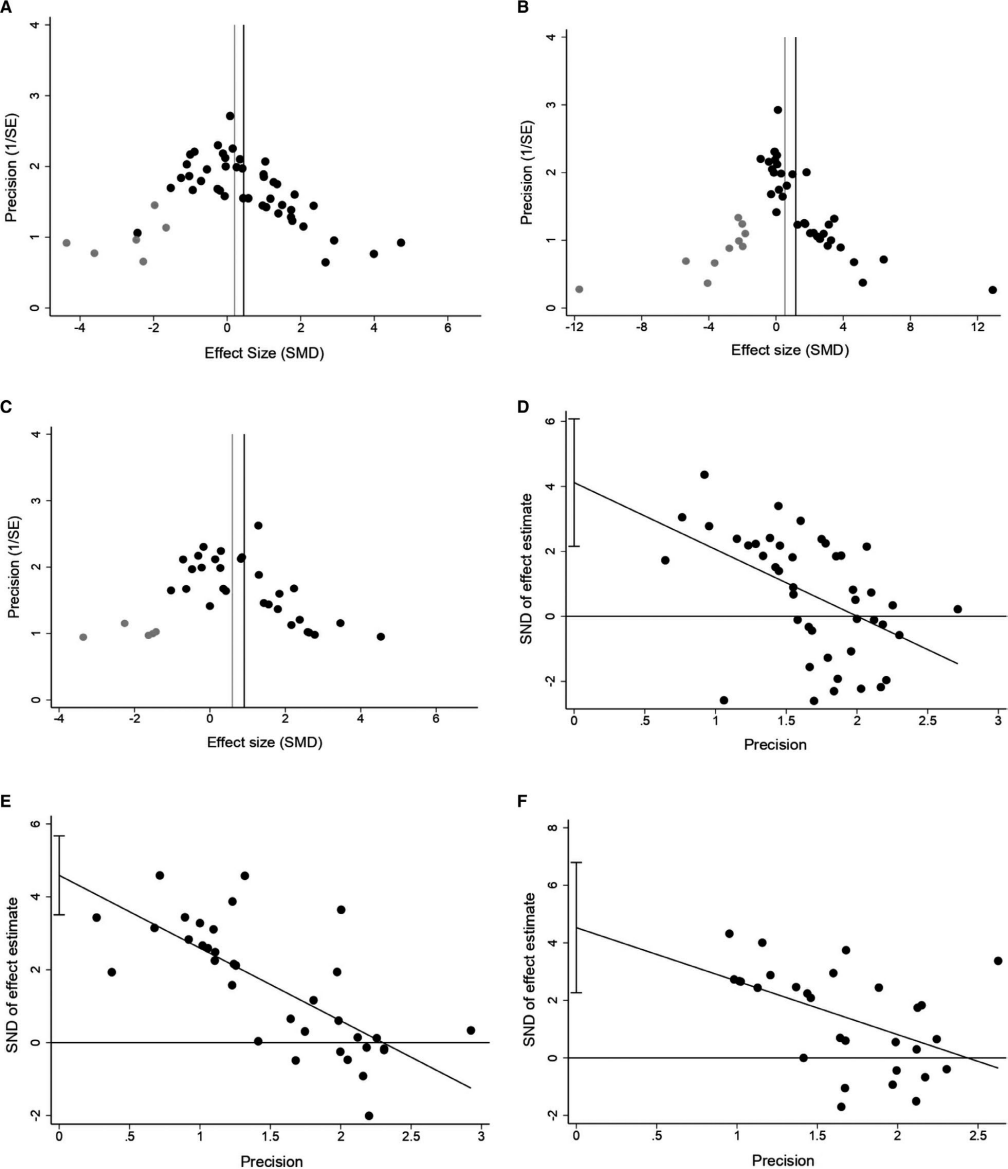
Publication bias assessment. Funnel plots for cerebral hemorrhage (**A**), infarct size (**B**), and neurobehavioral score (**C**), showing the distribution of published study outcomes (black circles) and additional missing outcomes (gray circles) imputed by trim and fill. The vertical black lines represent the actual estimate, and the gray vertical lines represent the theoretical estimate, when publication bias does not exist. Egger regression for cerebral hemorrhage (**D**), infarct size (**E**), and neurobehavioral score (**F**), confirming potential evidence for publication bias. The short vertical lines represent the 95% CI. SMD indicates standardized mean difference.

We performed funnel plots, trim and fill method, and the Egger test to assess the potential publication bias. Funnel plots showed minor asymmetry for cerebral hemorrhage and neurobehavioral outcome. But obvious asymmetry for infarct size was observed in the funnel plot (Figure S9). A subsequent trim and fill analysis suggested 6 theoretically missing studies (Figure 6A) with an adjusted improvement in cerebral hemorrhage of SMD of 0.2 (95% CI, –0.15 to 0.56; compared with SMD of 0.45 [95% CI, 0.11–0.78]). For infarct size, we estimate 10 unpublished articles (Figure 6B), giving an adjusted overall effect of SMD of 0.53 (95% CI, 0.03–1.03; compared with SMD of 1.18 [95% CI, 0.73–1.64]). We estimate 5 unpublished neurobehavioral outcomes (Figure 6C) with a corrected improvement of SMD of 0.59 (95% CI, 0.15–1.03; compared with SMD of 0.91 [95% CI, 0.49–1.32]). The Egger regression indicated significant publication bias for cerebral hemorrhage (*P*<0.001; Figure 6D), infarct size (*P*<0.001; Figure 6E), and neurobehavioral outcome (*P*<0.001; Figure 6F), again suggesting funnel plot asymmetry.

We also performed sensitivity analysis by removing one study at a time to confirm whether the results were robust. Sensitivity analysis showed that excluding any one study did not affect the results presented, suggesting the stability of our results (Figure S10).

## Discussion

In this meta‐analysis, we assessed the efficacy of antithrombotic agents on tPA‐induced HT in preclinical studies. The results confirmed the beneficial effects of antithrombotic agents in animal models to improve the cerebral hemorrhage, infarct size, and neurobehavioral outcome following tPA‐induced HT. The analysis also showed that antiplatelet agents and anticoagulant agents were the more effective subtypes of antithrombotic agents, whereas fibrinolytic agents produced no significant differences. Furthermore, our results indicated that study quality, random allocation, control of temperature, anesthetic used, stroke model used, route of drug delivery, time of drug administration, and time of assessment were significant factors that influenced the efficacy of antithrombotic agents.

Given the high risk of occurrence of a new ischemic event following an ischemic stroke, it is necessary to use antithrombotic agents to prevent recurrence.^36^ However, the benefits of stroke risk reduction have to be weighed against the risk of HT.^37^ In our study, antithrombotic therapy overall improved cerebral hemorrhage, infarct size, and neurobehavioral outcome in animal models of tPA‐induced HT. Specifically, antiplatelet agents revealed statistically significant effects in all the outcomes. Anticoagulant agents showed significant effects in infarct size and neurobehavioral score, whereas fibrinolytic agents did not show any significant improvement in all the outcomes. Our results were consistent with recent clinical studies. The RESTART (Restart or Stop Antithrombotics Randomized Trial) suggested that the risk of recurrent intracerebral hemorrhage is probably too small to exceed the found benefits of antiplatelet therapy for the secondary prevention of major vascular events.^38^ Another study showed that antiplatelet therapy before or during bridging thrombolysis in patients with acute ischemic stroke did not increase the risk of bleeding complications and had no impact on outcome.^39^ Similarly, anticoagulant therapy resumption after intracerebral hemorrhage decreased thromboembolic complications and long‐term mortality without significantly increasing bleeding complications.^40^ The RETRACE (German‐Wide Multicenter Analysis of Oral Anticoagulation Associated Intracerebral Hemorrhage) study showed that resumption of anticoagulant therapy was associated with lower risk of ischemic events without increased bleeding complications.^41^ A study provided evidence supporting the use of uridylyl phosphate adenosine, not tPA, for fibrinolytic therapy after intracerebral hemorrhage.^42^ Intravenous tPA has been shown to be associated with increased rates of intracerebral hemorrhage and length of stay without any improvement in clinical outcome.^43^

The study quality of the included studies was moderate. Only 1 study received a low score of 2, and 3 studies received a relatively high score of 9, with 1 substudy of these even receiving a full score of 10. Study quality criteria, such as allocation concealment, use of comorbid animals, and sample size calculation, were not reported commonly. We found a statistically significant correlation between study quality and year of publication. This finding was in line with previous studies that suggested better compliance with study quality criteria over time.^44^ Furthermore, we also noted that studies with a low study quality score were more likely to have large effect size. This suggested low study quality might be associated with an overestimation of effect size.^45^ Consistent with previous findings, randomization to group was significantly associated with a lower improvement in outcomes.^9^ Studies that blinded assessment of outcome reported lower effect size in cerebral hemorrhage, supporting the results of previous meta‐analyses.^45^ However, studies that reported blinded outcome assessment revealed significantly larger improvements in neurobehavioral score. This paradox may be attributable to the confounding effect of other variables. Given that failure to blind assessment and failure to randomize study subjects might lead to overestimation of effect sizes, we strongly recommend implementation of standard methods in preclinical studies.^46^

Only 8 publications (36%) reported a sample size calculation. Previous studies also indicated that sample size calculations in animal studies in neuroscience were rarely reported.^47^ In our study, the lower effects were seen in studies that performed a sample size calculation. Studies that performed temperature control during induction of stroke reported significantly larger improvement. This was probably because good nursing during surgery promoted the rehabilitation of animals.

Our results indicated that the use of ketamine anesthesia at stroke induction was associated with larger effects, whereas chloral hydrate was associated with lower effects. This was because ketamine had neuroprotective properties,^48^ whereas chloral hydrate might have potentially neurotoxic effects.^49^, ^50^ In addition, the use of isoflurane was also associated with good outcome in our study. This phenomenon is understandable as neuroprotection provided by isoflurane has been observed in stroke models by many studies.^51^, ^52^ Two stroke models were included in this analysis: embolic stroke model and transient middle cerebral artery occlusion model. Studies that used embolic stroke model reported larger improvement in infarct size and neurobehavioral score compared with studies that used transient occlusion model. This may be partly because the transient middle cerebral artery occlusion model usually led to more severe brain damage.^46^

Efficacy was not significantly different in studies that used different animal species (mainly included rats and mice) to establish stroke models. The larger effects were seen in studies that used intravenous delivery, intracerebroventricular delivery, and combined delivery. This was probably because these routes of drug delivery were more readily absorbed compared with intraperitoneal delivery and oral gavage delivery.^53^

We also observed that time of drug administration had significant impact on all the outcomes. The larger improvements were seen in studies that reported first administration 3 hours after stroke onset. Moreover, studies that reported pretreatment with antithrombotic agents revealed lower effects compared with posttreatment. This was consistent with clinical findings that antithrombotic therapy was not recommended when the risk of bleeding was high and should be reintroduced following a primary HT.^40^ The largest effects were seen in studies reporting assessment time >40 hours after stroke onset. This could be because of the enhanced self‐healing ability of animals over time. Many evaluated factors were correlated in this study, such as randomization and temperature control (data not shown). So, results of the meta‐regression/subgroup analyses need to be interpreted cautiously.

Considerable asymmetry was found in funnel plots, suggesting the presence of publication bias in this study. The results were further confirmed by the Egger test. This indicated that the overall efficacy could be overestimated because of lack of null or negative studies.^46^ Using trim and fill method, no statistically significant improvement was found in cerebral hemorrhage after 6 theoretically missing studies were included. However, a significant improvement in infarct size and neurobehavioral score remained after trim and fill. Only English‐language articles were included in this analysis, which may partly account for the publication bias.

### Limitations

This study has several limitations. Studies that only quantified the incidence of HT were excluded because of the small number of such studies, which may also lead to publication bias. Only 1 of 44 included studies reported the use of female animals, which meant that the effects of sex role could not be assessed. Where multiple doses of a drug were given, only the dose with the best efficacy was included, which may lead to bias. Fibrinolytic agents were not used to assess neurobehavioral score, so the efficacy of fibrinolytic therapy in neurobehavioral outcome remained unknown. Then, efficacy of individual drugs could not evaluated because of the lack of sufficient studies. It was also unpractical to include all the factors in one model because of limited number of studies.

## Conclusions

To the best of our knowledge, this is the first systematic review and meta‐analysis that evaluated the efficacy of antithrombotic agents on tPA‐induced HT in animal models. We concluded that antithrombotic therapy, especially antiplatelet therapy, resulted in improvement of cerebral hemorrhage, infarct size, and neurobehavioral outcome in animal models of tPA‐induced HT. Furthermore, we discovered key factors that influenced the effects of antithrombotic agents through meta‐regression and subgroup analyses. Our results provided some guidance for future animal studies investigating the efficacy of antithrombotic agents on tPA‐induced HT and valuable insight into clinical drug use and trial design. We should interpret the results with caution because of the observed heterogeneity and publication bias.

## Sources of Funding

This study was supported by the National Natural Science Foundation of China (Grant No. 81903942) and the China Postdoctoral Science Foundation (Grant No. 2019M650393).

## Disclosures

None.

**Table 1:** Characteristics of Included Studies

Author	Year	Intervention	Mechanism	Route	Dose of Agent	Time of Administration, min	Species	Sex	Stroke Model	Dose of tPA, mg/kg	Time of tPA Administration, min
Andreou, AP^14^	2015	APC	Inactivation of factors Va and VIIIa	IV	250 μg/kg	180	Mouse	Men	TMM	10	180
		APC (36–39)	APC variant reduced anticoagulant activity	IV	250 μg/kg	180	Mouse	Men	TMM	10	180
Cheng, T^15^	2006	APC	Inactivation of factors Va and VIIIa	IV	0.02 mg/kg	35	Mouse	Unclear	TMM	10	35
				IV	0.04 mg/kg	35	Mouse	Unclear	TMM	10	35
				IV	0.2 mg/kg	35	Mouse	Unclear	TMM	10	35
				IV	2 mg/kg	215	Mouse	Unclear	TMM	10	35
				IV	0.4 mg/kg	240	Rat	Men	ESM	10	240
Gautier, S^16^	2003	TLP	Thrombus lysis product	IV	Unclear	360	Rat	Men	TMM	10	360
Goebel, S^17^	2013	Revacept	GPVI‐Fc fusion protein	IV	1 mg/kg	90	Mouse	Men	TMM	10	90
Hase, Y^18^	2012	Cilostazol	Phosphodiesterase inhibitor	ID	0.3%	−10 080	Mouse	Men	TMM	10	45
				ID	0.3%	−10 080	Mouse	Men	TMM	10	90
Houng, AK^19^	2014	4H9	Anti–α2‐antiplasmin antibody	IV	9.3 or 21.3 mg/kg	150	Mouse	Men	ESM	2	150
				IV	9.3 or 21.3 mg/kg	150	Mouse	Men	ESM	10	150
Huang, Y^20^	2018	SMTP‐7	Enhance both activation and fibrin binding of plasminogen	IV	10 mg/kg	60	Mouse	Men	TMM	10	60
Ishiguro, M^21^	2010	Cilostazol	Phosphodiesterase inhibitor	IP	10 mg/kg	360	Mouse	Men	TMM	10	360
Izuma, H^22^	2018	Rivaroxaban	Factor Xa inhibitor	Gavage	10 mg/kg	180	Rat	Men	TMM	10	270
				Gavage	20 mg/kg	180	Rat	Men	TMM	10	270
Kasahara, Y^23^	2012	Cilostazol	Phosphodiesterase inhibitor	ID	0.3%	−10 080	Mouse	Men	TMM	10	90
				ID	0.3%	−10 080	Mouse	Men	TMM	10	120
				ID	0.3%	−10 080	Mouse	Men	TMM	10	180
				ID	0.3%	−10 080	Mouse	Men	TMM	10	240
		Aspirin	Thromboxane A2‐synthase inhibitor	ID	0.1%	−10 080	Mouse	Men	TMM	10	90
Kono, S^24^	2014	Warfarin	Vitamin K antagonist	Gavage	0.2 mg/kg	−10 080	Rat	Men	TMM	10	120
		Rivaroxaban	Factor Xa inhibitor	Gavage	2 mg/kg	−10 080	Rat	Men	TMM	10	120
		Apixaban	Factor Xa inhibitor	Gavage	10 mg/kg	−10 080	Rat	Men	TMM	10	120
Lapchak, PA^25^	2002	SM‐20302	Glycoprotein IIb/IIIa receptor antagonist	IV	5 mg/kg	65	Rabbit	Men	ESM	3.3	65
Li, Q^26^	2017	BAY 60‐6583	Adenosine A2b receptor agonist	IV	1 mg/kg	120	Rat	Men	TMM	10	120
Momi, S^27^	2013	ALX‐0081	vWF inhibitor	IV, SC	5 mg/kg	0	Guinea pig	Men	TMM	0.608	60
Pfeilschifter, W^28^	2011	Warfarin	Vitamin K antagonist	ID	2 mg/kg	−1440	Mouse	Men	TMM	10	180
Ploen, R^29^	2014	Rivaroxaban	Factor Xa inhibitor	Gavage	30 mg/kg	−60	Mouse	Men	TMM	9	105
				Gavage	30 mg/kg	−60	Mouse	Men	TMM	9	165
				Gavage	30 mg/kg	−60	Rat	Men	ESM	9	120
		Warfarin	Vitamin K antagonist	ID	0.4 mg/kg	−4320	Mouse	Men	TMM	9	105
				ID	0.4 mg/kg	−4320	Mouse	Men	TMM	9	165
				ID	INR 2 to 3	−4320	Rat	Men	ESM	9	120
Schuhmann, MK^30^	2019	JAQ1	Anti‐GPVI antibody	IP	100 μg	−7200	Mouse	Men	TMM	10	60
Wang, Y^31^	2012	3K3A‐APC	APC analog with reduced anticoagulant activity	IV, IP	2 mg/kg	240	Mouse	Men	TMM	10	240
				IV, IP	2 mg/kg	240	Mouse	Men	TMM	10	240
				IV	2 mg/kg	240	Rat	Men	ESM	10	240
Wang, Y^32^	2013	3K3A‐APC	APC analog with reduced anticoagulant activity	IV, IP IV	0.2 mg/kg 0.2 mg/kg	240 240	Mouse Rat	Women Men	TMM ESM	10 10	240 240
Wang, L^33^	2013	rADAMTS13	vWF‐cleaving protease	ICV	100 ng	180	Mouse	Men	TMM	10	120
Zheng, Y^34^	2019	Aspirin and clopidogrel	Thromboxane A2‐synthase inhibitor and P2Y12 receptor antagonist	ID	Aspirin 0.4 mg/mL, clopidogrel 0.15 mg/mL	−4320	Mouse	Men	TMM	10	120
				ID	Aspirin 0.4 mg/mL, clopidogrel 0.15 mg/mL	−4320	Mouse	Men	TMM	6.67	120
Zlokovic, BV^35^	2005	APC	Inactivation of factors Va and VIIIa	IV	0.2 mg/kg	240	Rat	Men	ESM	10	240
				IV	0.2 mg/kg	240	Mouse	Men	ESM	10	240

APC indicates activated protein C; ESM, embolic stroke model; GPVI, glycoprotein VI; ID, in diet; INR, international normalized ratio; rADAMTS13, recombinant ADAMTS13; SMTP‐7, Stachybotrys microspora triprenyl phenol‐7; TLP, thrombolysis product; TMM, transient middle cerebral artery occlusion model; tPA, tissue‐type plasminogen activator; and vWF, von Willebrand factor.

## Supporting information


**Data S1** **Tables S1–S2** **Figures S1–S10**
