# Supplementary material for: Antithrombotic Agents for tPA‐Induced Cerebral Hemorrhage: A Systematic Review and Meta‐Analysis of Preclinical Studies
Source: J Am Heart Assoc. 2020 Dec 5;9(24):e017876. doi: 10.1161/JAHA.120.017876 (PMC7955384; doi:10.1161/JAHA.120.017876)
Supplement: Supplementary file 1 — Data S1 Tables S1–S2 Figures S1–S10 [file JAH3-9-e017876-s001.pdf]

SUPPLEMENTAL MATERIAL

Data S1. Search strategy.

1. Search strategy used in PubMed database.

#1. tPA OR rtPA OR t-PA OR rt-PA OR tissue plasminogen activator OR tissue-plasminogen activator OR alteplase[Title/Abstract] Items found: 50269
#2. hemorrhagic transformation OR hemorrhage OR haemorrhage[Title/Abstract] Items found: 409389
#3. stroke OR ischemia OR cerebral[Title/Abstract] Items found: 977874
#4. #1 AND #2 AND #3 Items found: 3701
#5. #4 NOT (review[Publication Type]) Items found: 3076
#6. #5 NOT (clinical trial[Publication Type]) Items found: 2548
#7. #6 NOT (meta-analysis[Publication Type]) Items found: 2495
#8. #7 NOT (patient[Title]) Items found: 2430
#9. #8 NOT (patients[Title]) Items found: 1915
#10. #9 NOT (trial[Title]) Items found: 1888
#11. #10 NOT (review[Title]) Items found: 1871
#12. #11 NOT (meta-analysis[Title]) Items found: 1866
Final Result: 1866 (By April 20, 2020)

2. Search strategy used in Web of Science database.

#1. TS=(tPA OR rtPA OR t-PA OR rt-PA OR tissue plasminogen activator OR tissue-plasminogen activator OR alteplase) Items found: 98589
#2. TS=(hemorrhagic transformation OR hemorrhage OR haemorrhage) Items found: 451024
#3. TS=(stroke OR ischemia OR cerebral) Items found: 1792729
#4. #1 AND #2 AND #3 Items found: 6853
#5. #4 NOT TI=(patient) Items found: 5444
#6. #5 NOT TI=(patients) Items found: 5444
#7. #6 NOT TI=(trial) Items found: 5076
#8. #7 NOT TI=(review) Items found: 4873
#9. #8 NOT TI=(meta-analysis) Items found: 4822
#10. #9 AND Refine: Publication Type: (ARTICLE) Items found: 4149
#11. #10 AND Refine: Language: (ENGLISH) Items found: 3857
Final Result: 3857 (By April 20, 2020)

3. Search strategy used in Scopus database.

#1. TITLE-ABS-KEY(tPA OR rtPA OR t-PA OR rt-PA OR tissue plasminogen activator OR tissue-plasminogen activator OR alteplase) Items found: 47295
#2. TITLE-ABS-KEY(hemorrhagic transformation OR hemorrhage OR haemorrhage)

Items found: 39374

#3. TITLE-ABS-KEY(stroke OR ischemia OR cerebral)   Items found: 1222057

#4. #1 AND #2 AND #3   Items found: 1150

#5. #4 AND NOT TITLE (patient)   Items found: 958

#6. #5 AND NOT TITLE (patients)   Items found: 958

#7. #6 AND NOT TITLE (trial)   Items found: 921

#8. #7 AND NOT TITLE (review)   Items found: 898

#9. #8 AND NOT TITLE (meta-analysis)   Items found: 894

#10. #9 AND (LIMIT-TO (DOCTYPE,"ar"))   Items found: 674

#11. #10 AND (LIMIT-TO (LANGUAGE,"English"))   Items found: 625

Final Result: 625 (By April 20, 2020)

Table S1. Study quality score report.

Author	Year	(1)	(2)	(3)	(4)	(5)	(6)	(7)	(8)	(9)	(10)	Aggregate quality score
Andreou, A	2015	+	+	+		+	+			+	+	7
Cheng, T	2006a/b	+	+				-/+			+	+	4/5
Gautier, S	2003	+	+			+	+	+		+		6
Goebel, S	2013	+				+	+			+	+	5
Hase, Y	2012	+	+			+	+			+	+	6
Houng, A	2014	+	+			+	+		+	+		6
Huang, Y	2018	+	+				+			+	+	5
Ishiguro, M	2010	+	+	+	+		+			+	+	7
Izuma, H	2018	+					+	+		+		4
Kasahara, Y	2012	+	+			+	+			+	+	6
Kono, S	2014	+	+				+			+	+	5
Lapchak, P	2002	+		+		+	+		+	+		6

Li, Q	2017	+	+	+	+	+	+		+	+	+	9
Momi, S	2013	+	+							+	+	4
Pfeilschifter, W	2011	+		+	+		+		+	+	+	7
Ploen, R	2014	+		+		+	+		+	+	+	7
Schuhmann, M	2019	+				+			+	+	+	5
Wang, Y	2012a/b	+	+				-/+			+	+	4/5
Wang, Y	2013a/b	+	+	+	+	+	-/+	+	+	+	+	9/10
Wang, L	2013	+	+			+	+	+		+	+	7
Zheng, Y	2019	+	+	+	+	+	+		+	+	+	9
Zlokovic, B	2005a/b	+	+				-/+					2/3

The lowercase letter “a” indicated that mice were used in this study, the lowercase letter “b” indicated that rats were used in this study.

(1) Publication in a peer-reviewed journal
(2) Control of temperature
(3) Random allocation to groups
(4) Allocation concealment (blinded induction of ischemia)
(5) Blinded assessment of outcome
(6) Use of an anesthetic without intrinsic neuroprotective activity (ketamine)
(7) The use of co-morbid animals
(8) Performing a sample size calculation
(9) Compliance with animal welfare regulations
(10) Statement of potential conflicts of interest

Table S2. Detection methods used in each study.

Author	Year	Intervention	Characteristics (Dose of agent, Species, Dose of tPA ,Time of tPA)	Assessment of cerebral hemorrhage	Assessment of infarct size	Assessment of neurobehavioral outcomes
Andreou, A	2015	APC	250 μg/kg, Mouse, 10 mg/kg, 180 min	Hemoglobin content	Infarct volume	Neurological score
		APC(36-39)	250 μg/kg, Mouse, 10 mg/kg, 180 min			
Cheng, T	2006	APC	0.2 mg/kg, Mouse, 10 mg/kg, 35 min	Hemoglobin content	Infarct volume	
		APC	2 mg/kg, Mouse, 10 mg/kg, 35 min	Hemorrhage area		
		APC	0.4 mg/kg, Rat, 10 mg/kg, 240 min			Neurological severity score
Gautier, S	2003	TLP	Unclear, Rat, 10 mg/kg, 360 min	Hemorrhage score	Infarct volume	
Goebel, S	2013	Revacept	1 mg/kg, Mouse, 10 mg/kg, 90 min	Hemoglobin content	Infarct volume	
Hase, Y	2012	Cilostazol	0.3%, Mouse, 10 mg/kg, 45 min	Hemorrhage volume	Infarct volume	Neurological deficit score
		Cilostazol	0.3%, Mouse, 10 mg/kg, 90 min			
Houng, A	2014	4H9	9.3 or 21.3 mg/kg, Mouse, 2 mg/kg, 150 min	Hemorrhage volume	Infarct volume	
		4H9	9.3 or 21.3 mg/kg, Mouse, 10 mg/kg, 150 min			
Huang, Y	2018	SMTP-7	10 mg/kg, Mouse, 10 mg/kg, 60 min	Hemorrhage area		
Ishiguro, M	2010	Cilostazol	10 mg/kg, Mouse, 10 mg/kg, 360 min	Hemoglobin content	Infarct volume	Neurological score
Izuma, H	2018	Rivaroxaban	10 mg/kg, Rat, 10 mg/kg, 270 min	Hemoglobin content	Infarct volume	Neurological score
		Rivaroxaban	20 mg/kg, Rat, 10 mg/kg, 270 min			
Kasahara, Y	2012	Cilostazol	0.3%, Mouse, 10 mg/kg, 90 min	Hemorrhage score		
		Cilostazol	0.3%, Mouse, 10 mg/kg, 120 min			

		Cilostazol	0.3%, Mouse, 10 mg/kg, 180 min	Hemorrhage score		
		Cilostazol	0.3%, Mouse, 10 mg/kg, 240 min			
		Aspirin	0.1%, Mouse, 10 mg/kg, 90 min			
Kono, S	2014	Warfarin	0.2 mg/kg, Rat, 10 mg/kg, 120 min	Hemorrhage volume	Infarct volume	Paraparesis score
		Rivaroxaban	2 mg/kg, Rat, 10 mg/kg, 120 min			
		Apixaban	10 mg/kg, Rat, 10 mg/kg, 120 min			
Lapchak, P	2002	SM-20302	5 mg/kg, Rabbit, 3.3 mg/kg, 65 min	Hemorrhage score	Infarct score	
Li, Q	2017	BAY 60-6583	1 mg/kg, Rat, 10 mg/kg, 120 min	Hemoglobin content	Infarct volume	Neurologic deficit score
Momi, S	2013	ALX-0081	5 mg/kg, Guinea pig, 0.608 mg/kg, 60 min	Hemoglobin content	Infarct area	
Pfeilschifter, W	2011	Warfarin	2 mg/kg, Mouse, 10 mg/kg, 180 min	Hemoglobin content		Neurologic deficit score
Ploen, R	2014	Rivaroxaban	30 mg/kg, Mouse, 9 mg/kg, 105 min	Hemoglobin content	Infarct volume	Neurologic score
		Rivaroxaban	30 mg/kg, Mouse, 9 mg/kg, 165 min	Hemorrhage score		
		Rivaroxaban	30 mg/kg, Rat, 9 mg/kg, 120 min			
		Rivaroxaban	30 mg/kg, Mouse, 9 mg/kg, 105 min	Hemorrhage score		
		Warfarin	30 mg/kg, Mouse, 9 mg/kg, 105 min	Hemoglobin content	Infarct volume	Neurologic score
		Warfarin	30 mg/kg, Mouse, 9 mg/kg, 165 min	Hemorrhage score		
		Warfarin	30 mg/kg, Rat, 9 mg/kg, 120 min			
		Warfarin	30 mg/kg, Mouse, 9 mg/kg, 105 min	Hemorrhage score		
Schuhmann, M	2019	JAQ1	100 µg, Mouse, 10 mg/kg, 60 min	Hemorrhage score	Infarct volume	Mann-Whitney test
Wang, Y	2012	3K3A-APC	2 mg/kg, Mouse, 10 mg/kg, 240 min	Hemoglobin content	Infarct volume	Motor neurological score
		3K3A-APC	2 mg/kg, Mouse, 10 mg/kg, 240 min			Foot-fault test
		3K3A-APC	2 mg/kg, Rat, 10 mg/kg, 240 min			Modified Neurological Severity Score (mNSS)

Wang, Y	2013	3K3A-APC	0.2 mg/kg, Mouse, 10 mg/kg, 240 min	Hemoglobin content	Infarct volume	Foot-fault test
		3K3A-APC	0.2 mg/kg, Rat, 10 mg/kg, 240 min	Hemorrhage area		Modified Neurological Severity Score (mNSS)
Wang, L	2013	rADAMTS13	100 ng, Mouse, 10 mg/kg, 120 min	Hemoglobin content		Neurologic score
Zheng, Y	2019	Aspirin and Clopidogrel	As 0.4 mg/mL, Cl 0.15 mg/mL, Mouse, 10 mg/kg, 120 min	Hemorrhage area	Infarct volume	
		Aspirin and Clopidogrel	As 0.4 mg/mL, Cl 0.15 mg/mL, Mouse, 6.67 mg/kg, 120 min			
Zlokovic, B	2005	APC	0.2 mg/kg, Rat, 10 mg/kg, 240 min	Hemorrhage area	Infarct volume	Neurological severity score
		APC	0.2 mg/kg, Mouse, 10 mg/kg, 240 min			Motor neurological score

Admin, administration; APC, activated protein C; TLP, thrombolysis products; SMTP-7, Stachybotrys microspora triprenyl phenol-7.

Figure S1. Forest plot of antithrombotic agents on cerebral hemorrhage.

Study or Subgroup	tPA Mean	tPA SD	tPA Total	Antithrombotic Mean	Antithrombotic SD	Antithrombotic Total	Weight	Std. Mean Difference IV, Random, 95% CI
**1.1.1 APC**								
APC[14]	1.73	0.38	4	1.46	0.45	7	2.2%	0.58 [-0.69, 1.84]
APC(b)[15]	0.69	0.21	3	0.14	0.1	3	0.9%	2.68 [-0.36, 5.71]
APC(c)[15]	128.7	85.19	6	10.43	24.62	6	2.1%	1.74 [0.33, 3.16]
APC(d)[15]	90.58	54.99	6	21.68	53.1	6	2.2%	1.18 [-0.09, 2.45]
APC(e)[35]	90.3	62.79	8	18.6	32.24	8	2.4%	1.36 [0.24, 2.48]
**Subtotal (95% CI)**			**27**			**30**	**9.9%**	**1.26 [0.64, 1.87]**
Heterogeneity: Tau² = 0.00; Chi² = 2.44, df = 4 (P = 0.65); I² = 0%								
Test for overall effect: Z = 4.01 (P < 0.0001)								
**1.1.2 Cilostazol**								
Cilostazol[18]	3.78	3.29	5	0.19	0.14	5	2.0%	1.39 [-0.07, 2.86]
Cilostazol(b)[18]	0.78	0.91	5	0.09	0.07	5	2.1%	0.97 [-0.39, 2.32]
Cilostazol(c)[21]	582.93	269.9	10	332.32	187.37	7	2.5%	0.99 [-0.05, 2.03]
Cilostazol(d)[23]	1.39	0.51	8	0.4	0.51	8	2.3%	1.84 [0.61, 3.06]
Cilostazol(e)[23]	1.39	0.91	8	0.39	0.54	8	2.4%	1.26 [0.16, 2.37]
Cilostazol(f)[23]	2.01	1.1	8	1.02	0.74	8	2.5%	1.00 [-0.06, 2.06]
Cilostazol(g)[23]	2.26	0.88	8	1.88	0.85	8	2.6%	0.42 [-0.58, 1.41]
**Subtotal (95% CI)**			**52**			**49**	**16.5%**	**1.06 [0.63, 1.50]**
Heterogeneity: Tau² = 0.00; Chi² = 3.54, df = 6 (P = 0.74); I² = 0%								
Test for overall effect: Z = 4.81 (P < 0.00001)								
**1.1.3 4H9**								
4H9[19]	4.06	2.84	5	0.11	0.2	5	1.9%	1.77 [0.18, 3.36]
4H9(b)[19]	0.51	0.89	5	0.18	0.38	5	2.2%	0.44 [-0.83, 1.70]
**Subtotal (95% CI)**			**10**			**10**	**4.1%**	**1.01 [-0.28, 2.31]**
Heterogeneity: Tau² = 0.36; Chi² = 1.66, df = 1 (P = 0.20); I² = 40%								
Test for overall effect: Z = 1.53 (P = 0.13)								
**1.1.4 Rivaroxaban**								
Rivaroxaban[22]	26.5	5.4	8	26.8	8.7	8	2.6%	-0.04 [-1.02, 0.94]
Rivaroxaban(b)[24]	0.04	0.04	4	0.05	0.05	9	2.3%	-0.20 [-1.38, 0.99]
Rivaroxaban(c)[29]	0.28	0.52	6	0.68	0.76	12	2.6%	-0.55 [-1.55, 0.45]
Rivaroxaban(d)[29]	1.5	0.7	8	1.4	0.6	14	2.7%	0.15 [-0.72, 1.02]
Rivaroxaban(e)[29]	1.7	0.7	8	1.9	0.8	16	2.7%	-0.25 [-1.10, 0.60]
Rivaroxaban(f)[29]	1	1.1	7	1.1	0.7	15	2.7%	-0.11 [-1.01, 0.78]
**Subtotal (95% CI)**			**41**			**74**	**15.6%**	**-0.15 [-0.54, 0.23]**
Heterogeneity: Tau² = 0.00; Chi² = 1.19, df = 5 (P = 0.95); I² = 0%								
Test for overall effect: Z = 0.77 (P = 0.44)								
**1.1.5 Warfarin**								
Warfarin[24]	0.04	0.04	4	1.38	1.52	13	2.4%	-0.94 [-2.11, 0.24]
Warfarin(b)[28]	2.8	1	5	9.2	3.2	5	1.6%	-2.44 [-4.29, -0.59]
Warfarin(c)[29]	0.28	0.52	6	2.91	2.91	12	2.5%	-1.03 [-2.08, 0.02]
Warfarin(d)[29]	1.5	0.7	8	2.3	0.8	16	2.7%	-1.00 [-1.91, -0.10]
Warfarin(e)[29]	1.7	0.7	8	3.1	1	8	2.4%	-1.53 [-2.69, -0.38]
Warfarin(f)[29]	1	1.1	7	2.1	0.9	15	2.6%	-1.10 [-2.06, -0.13]
**Subtotal (95% CI)**			**38**			**69**	**14.1%**	**-1.18 [-1.63, -0.73]**
Heterogeneity: Tau² = 0.00; Chi² = 2.56, df = 5 (P = 0.77); I² = 0%								
Test for overall effect: Z = 5.17 (P < 0.00001)								
**1.1.6 3K3A-APC**								
3K3A-APC[31]	0.59	0.13	5	0.23	0.09	5	1.5%	2.91 [0.85, 4.96]
3K3A-APC(b)[31]	0.41	0.11	5	0.22	0.04	5	1.8%	2.07 [0.37, 3.78]
3K3A-APC(c)[31]	98.99	75.07	10	33.84	39.94	10	2.6%	1.04 [0.09, 1.98]
3K3A-APC(d)[32]	0.43	0.05	5	0.23	0.04	5	1.1%	3.99 [1.42, 6.56]
3K3A-APC(e)[32]	57.75	10.7	8	13.52	6.48	8	1.4%	4.73 [2.60, 6.85]
**Subtotal (95% CI)**			**33**			**33**	**8.4%**	**2.73 [1.29, 4.17]**
Heterogeneity: Tau² = 1.80; Chi² = 13.35, df = 4 (P = 0.010); I² = 70%								
Test for overall effect: Z = 3.71 (P = 0.0002)								

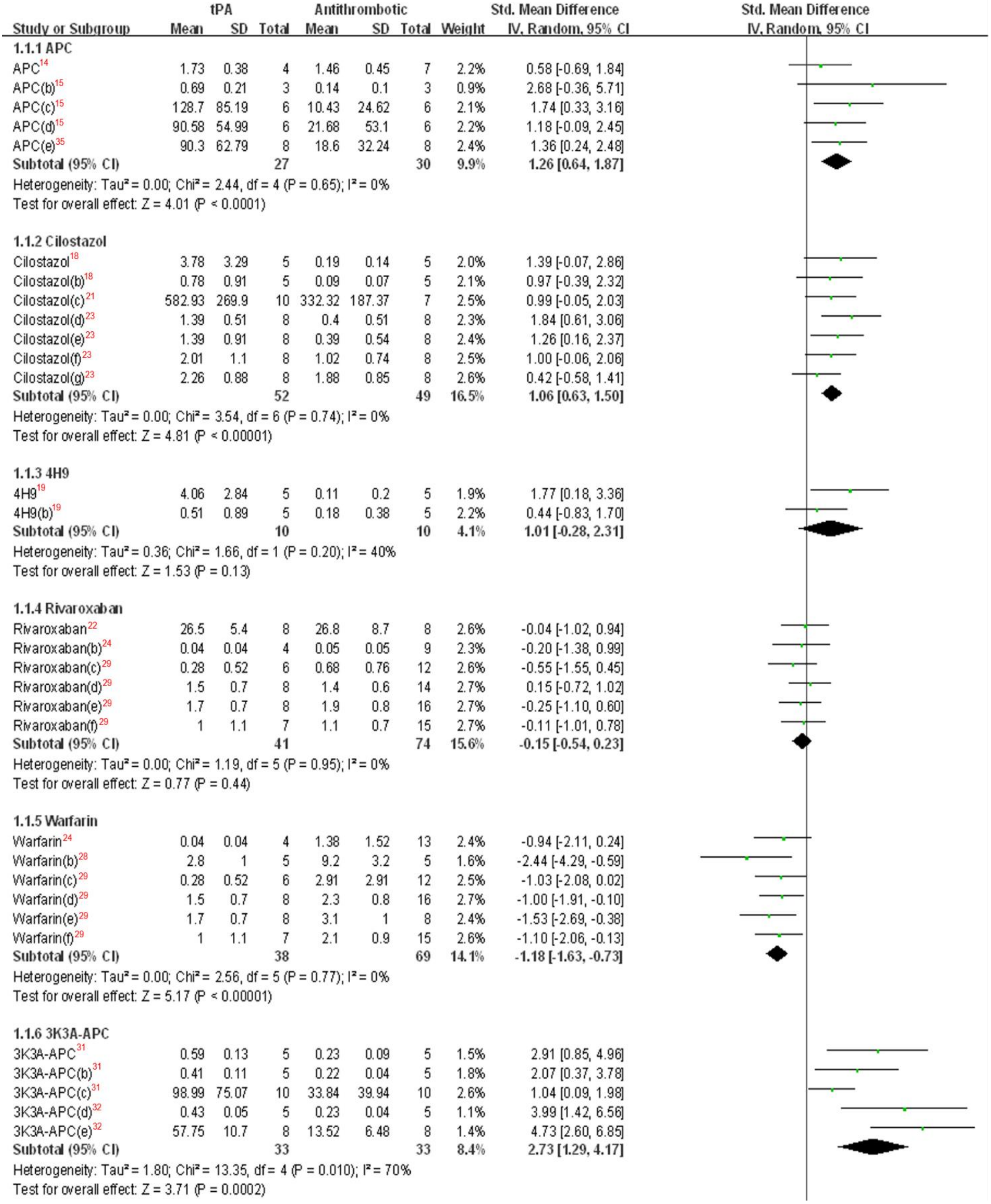

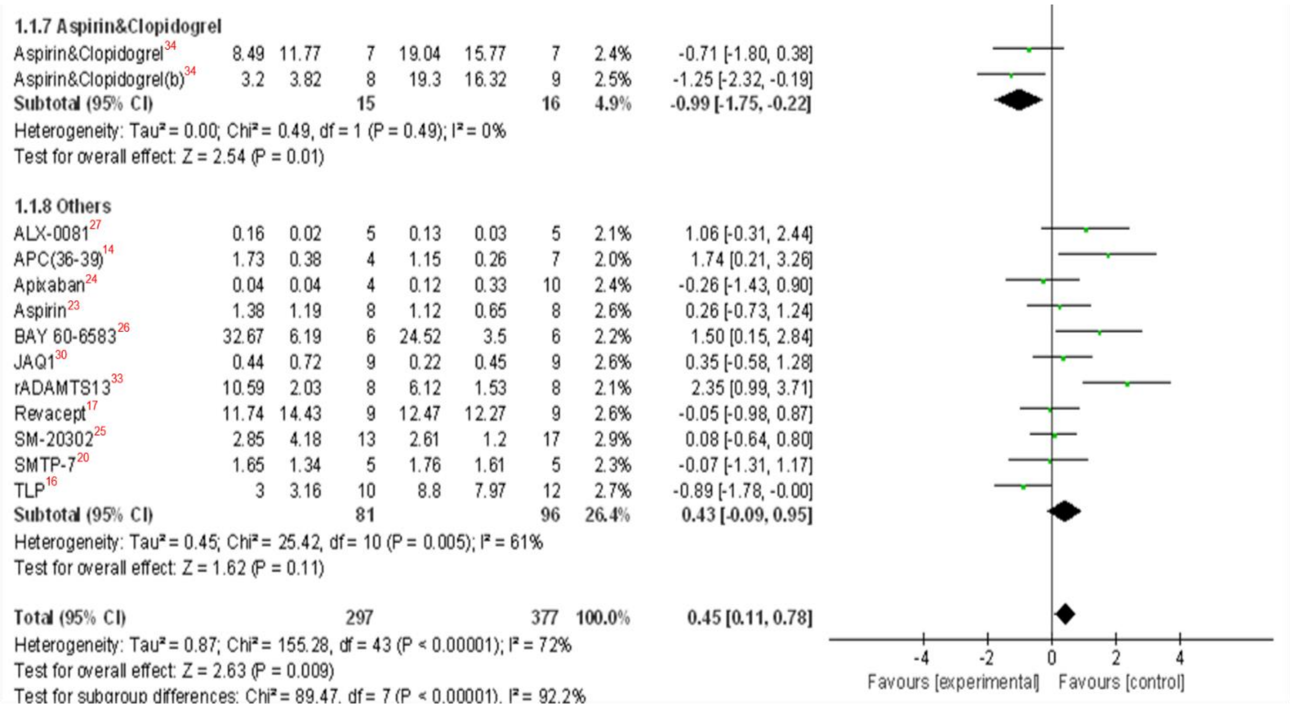

The horizontal lines indicate 95% confidence intervals for individual standardized mean differences (SMD), and the black diamonds indicates global SMD and confidence interval.

Figure S2. Forest plot of antithrombotic agents on infarct size.

Study or Subgroup	tPA Mean	tPA SD	tPA Total	Antithrombotic Mean	Antithrombotic SD	Antithrombotic Total	Weight	Std. Mean Difference IV, Random, 95% CI
**1.2.1 APC**								
APC[14]	35.38	5.98	3	23.66	8.84	6	2.8%	1.28 [-0.31, 2.88]
APC(b)[15]	61.64	9.02	3	12.88	5.7	3	0.6%	5.17 [-0.07, 10.41]
APC(c)[15]	38.92	5.27	6	18.28	7.91	6	2.5%	2.83 [1.05, 4.62]
APC(d)[15]	39.89	5.27	6	16.02	7.91	6	2.4%	3.28 [1.32, 5.24]
APC(e)[35]	40.04	6.53	8	21.35	4.53	8	2.8%	3.14 [1.55, 4.74]
APC(f)[35]	80.49	19.91	6	18.29	6.98	6	2.1%	3.85 [1.66, 6.04]
**Subtotal (95% CI)**			**32**			**35**	**13.2%**	**2.81 [1.97, 3.65]**
Heterogeneity: Tau² = 0.11; Chi² = 5.55, df = 5 (P = 0.35); I² = 10%								
Test for overall effect: Z = 6.54 (P < 0.00001)								
**1.2.2 Cilostazol**								
Cilostazol[18]	66.46	14.46	5	33.85	11.69	5	2.6%	2.24 [0.47, 4.01]
Cilostazol(b)[18]	71.08	18.15	5	42.15	12.31	5	2.8%	1.69 [0.12, 3.25]
Cilostazol(c)[21]	66.35	14.23	8	60.69	20.45	8	3.5%	0.30 [-0.68, 1.29]
**Subtotal (95% CI)**			**18**			**18**	**8.8%**	**1.24 [0.01, 2.47]**
Heterogeneity: Tau² = 0.66; Chi² = 4.54, df = 2 (P = 0.10); I² = 56%								
Test for overall effect: Z = 1.98 (P = 0.05)								
**1.2.3 Rivaroxaban**								
Rivaroxaban[22]	145.11	22.56	4	144.36	27.07	4	3.0%	0.03 [-1.36, 1.41]
Rivaroxaban(b)[24]	241.23	26.32	4	224.56	42.98	9	3.2%	0.40 [-0.80, 1.59]
Rivaroxaban(c)[29]	67.9	20.1	7	77.7	23	15	3.6%	-0.43 [-1.33, 0.48]
Rivaroxaban(d)[29]	78.9	14.1	8	78	16.8	14	3.6%	0.05 [-0.81, 0.92]
Rivaroxaban(e)[29]	318	63	8	322	47	16	3.6%	-0.07 [-0.92, 0.78]
**Subtotal (95% CI)**			**31**			**58**	**17.1%**	**-0.05 [-0.49, 0.39]**
Heterogeneity: Tau² = 0.00; Chi² = 1.26, df = 4 (P = 0.87); I² = 0%								
Test for overall effect: Z = 0.22 (P = 0.83)								
**1.2.4 Warfarin**								
Warfarin[24]	241.23	26.32	4	234.21	40.35	13	3.3%	0.18 [-0.95, 1.30]
Warfarin(b)[29]	67.9	20.1	7	69.1	18.4	15	3.6%	-0.06 [-0.96, 0.84]
Warfarin(c)[29]	78.9	14.1	8	80.2	14.4	16	3.6%	-0.09 [-0.94, 0.76]
Warfarin(d)[29]	318	63	8	325	41	8	3.5%	-0.12 [-1.11, 0.86]
**Subtotal (95% CI)**			**27**			**52**	**14.0%**	**-0.04 [-0.52, 0.43]**
Heterogeneity: Tau² = 0.00; Chi² = 0.18, df = 3 (P = 0.98); I² = 0%								
Test for overall effect: Z = 0.17 (P = 0.86)								
**1.2.5 4H9**								
4H9[19]	38.74	2.1	5	14.87	11.47	5	2.4%	2.61 [0.69, 4.54]
4H9(b)[19]	45.41	2.71	5	14.86	1.34	5	0.3%	12.91 [5.54, 20.28]
**Subtotal (95% CI)**			**10**			**10**	**2.7%**	**7.12 [-2.89, 17.13]**
Heterogeneity: Tau² = 45.42; Chi² = 7.01, df = 1 (P = 0.008); I² = 86%								
Test for overall effect: Z = 1.39 (P = 0.16)								
**1.2.6 3K3A-APC**								
3K3A-APC[31]	40.41	12.61	5	12.97	6.73	5	2.5%	2.45 [0.60, 4.31]
3K3A-APC(b)[31]	28.3	7.13	5	9.79	2.86	5	2.2%	3.08 [0.95, 5.21]
3K3A-APC(c)[31]	41.28	4.78	10	19.8	6.89	10	2.9%	3.47 [1.98, 4.96]
3K3A-APC(d)[32]	28.1	4.01	5	10.93	2.49	5	1.6%	4.65 [1.75, 7.54]
3K3A-APC(e)[32]	337.42	38.34	8	133.44	18.4	8	1.7%	6.41 [3.67, 9.15]
**Subtotal (95% CI)**			**33**			**33**	**10.7%**	**3.71 [2.53, 4.90]**
Heterogeneity: Tau² = 0.65; Chi² = 6.28, df = 4 (P = 0.18); I² = 36%								
Test for overall effect: Z = 6.15 (P < 0.00001)								
**1.2.7 Aspirin&Clopidogrel**								
Aspirin&Clopidogrel[34]	98.86	20.61	7	83.77	23.2	7	3.4%	0.64 [-0.44, 1.73]
Aspirin&Clopidogrel(b)[34]	93.12	20.25	8	99.44	30.33	9	3.5%	-0.23 [-1.19, 0.73]
**Subtotal (95% CI)**			**15**			**16**	**6.9%**	**0.17 [-0.68, 1.02]**
Heterogeneity: Tau² = 0.11; Chi² = 1.40, df = 1 (P = 0.24); I² = 29%								
Test for overall effect: Z = 0.39 (P = 0.70)								

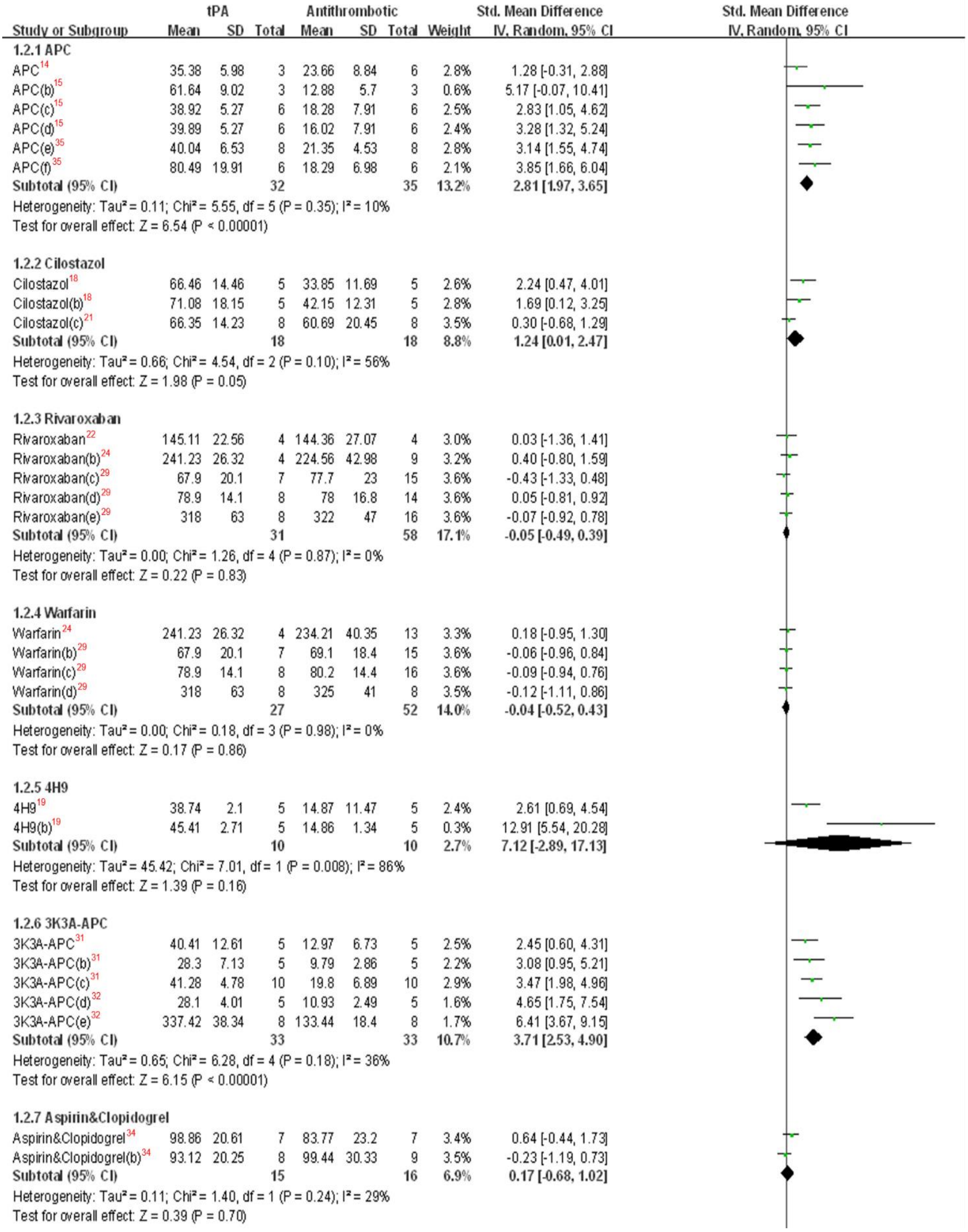

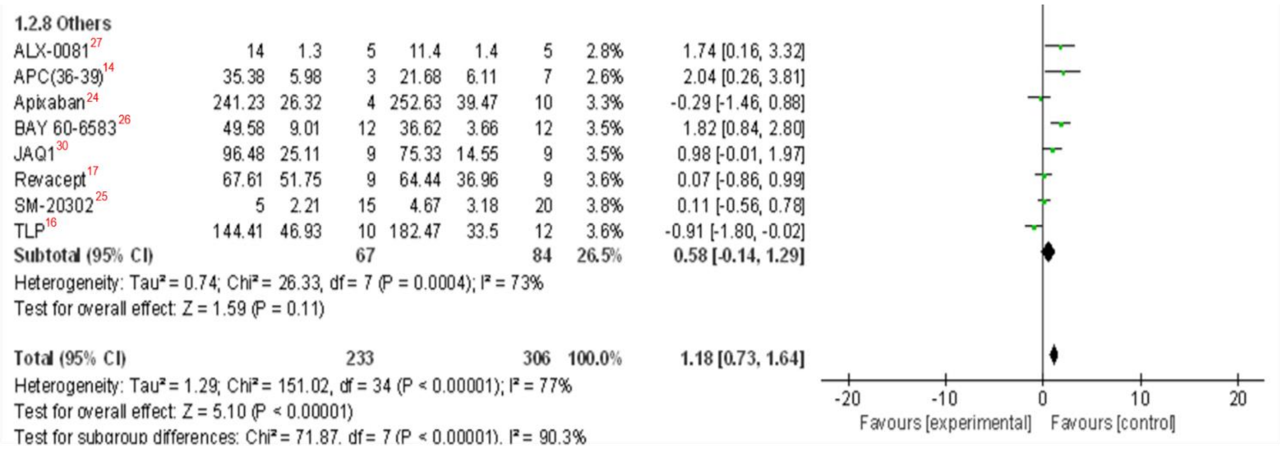

**1.2.8 Others**								
ALX-0081[27]	14	1.3	5	11.4	1.4	5	2.8%	1.74 [0.16, 3.32]
APC(36-39)[14]	35.38	5.98	3	21.68	6.11	7	2.6%	2.04 [0.26, 3.81]
Apixaban[24]	241.23	26.32	4	252.63	39.47	10	3.3%	-0.29 [-1.46, 0.88]
BAY 60-6583[26]	49.58	9.01	12	36.62	3.66	12	3.5%	1.82 [0.84, 2.80]
JAQ1[30]	96.48	25.11	9	75.33	14.55	9	3.5%	0.98 [-0.01, 1.97]
Revacept[17]	67.61	51.75	9	64.44	36.96	9	3.6%	0.07 [-0.86, 0.99]
SM-20302[25]	5	2.21	15	4.67	3.18	20	3.8%	0.11 [-0.56, 0.78]
TLP[16]	144.41	46.93	10	182.47	33.5	12	3.6%	-0.91 [-1.80, -0.02]
**Subtotal (95% CI)**			**67**			**84**	**26.5%**	**0.58 [-0.14, 1.29]**
Heterogeneity: Tau² = 0.74; Chi² = 26.33, df = 7 (P = 0.0004); I² = 73%								
Test for overall effect: Z = 1.59 (P = 0.11)								
**Total (95% CI)**			**233**			**306**	**100.0%**	**1.18 [0.73, 1.64]**
Heterogeneity: Tau² = 1.29; Chi² = 151.02, df = 34 (P < 0.00001); I² = 77%								
Test for overall effect: Z = 5.10 (P < 0.00001)								
Test for subgroup differences: Chi² = 71.87, df = 7 (P < 0.00001), I² = 90.3%								

The horizontal lines indicate 95% confidence intervals for individual standardized mean differences (SMD), and the black diamonds indicates global SMD and confidence interval.

Figure S3. Forest plot of antithrombotic agents on neurobehavioral score.

Study or Subgroup	tPA Mean	tPA SD	tPA Total	Antithrombotic Mean	Antithrombotic SD	Antithrombotic Total	Weight	Std. Mean Difference IV, Random, 95% CI
**1.3.1 APC**								
APC[14]	11.54	1.87	8	8.51	4.22	13	4.0%	0.82 [-0.10, 1.74]
APC(b)[15]	8.29	0.66	6	4.86	1.76	6	2.8%	2.38 [0.76, 4.00]
APC(c)[35]	8.38	1.5	8	5.84	1.07	8	3.5%	1.84 [0.62, 3.07]
APC(d)[35]	3.64	0.93	6	1.6	1.15	6	3.1%	1.80 [0.37, 3.23]
**Subtotal (95% CI)**			**28**			**33**	**13.5%**	**1.52 [0.83, 2.21]**
Heterogeneity: Tau² = 0.09; Chi² = 3.67, df = 3 (P = 0.30); I² = 18%								
Test for overall effect: Z = 4.34 (P < 0.0001)								
**1.3.2 Cilostazol**								
Cilostazol[18]	2.16	0.49	6	1.35	0.47	6	3.3%	1.56 [0.19, 2.92]
Cilostazol(b)[18]	2.58	0.23	5	1.78	0.32	5	2.4%	2.59 [0.68, 4.51]
Cilostazol(c)[21]	-1,166.27	237.07	5	-2,592.77	1,173.05	7	3.3%	1.43 [0.09, 2.77]
**Subtotal (95% CI)**			**16**			**18**	**9.0%**	**1.71 [0.86, 2.57]**
Heterogeneity: Tau² = 0.00; Chi² = 1.03, df = 2 (P = 0.60); I² = 0%								
Test for overall effect: Z = 3.92 (P < 0.0001)								
**1.3.3 Rivaroxaban**								
Rivaroxaban[22]	4.25	1.16	8	3.88	1.36	8	3.9%	0.28 [-0.71, 1.26]
Rivaroxaban(b)[22]	3	0.82	4	3	0.82	4	3.2%	0.00 [-1.39, 1.39]
Rivaroxaban(c)[24]	4.49	2.78	4	2.58	4.61	9	3.5%	0.42 [-0.77, 1.62]
Rivaroxaban(d)[29]	2.5	1.1	7	2.9	1.3	15	4.0%	-0.31 [-1.21, 0.59]
Rivaroxaban(e)[29]	3.2	1.9	8	2.7	1.5	14	4.1%	0.29 [-0.58, 1.17]
Rivaroxaban(f)[29]	2.9	1.6	6	3.3	1.8	12	3.9%	-0.22 [-1.20, 0.76]
**Subtotal (95% CI)**			**37**			**62**	**22.7%**	**0.06 [-0.35, 0.48]**
Heterogeneity: Tau² = 0.00; Chi² = 1.77, df = 5 (P = 0.88); I² = 0%								
Test for overall effect: Z = 0.29 (P = 0.77)								
**1.3.4 Warfarin**								
Warfarin[24]	4.49	2.78	4	6.61	1.68	13	3.6%	-1.03 [-2.22, 0.16]
Warfarin(b)[28]	4.5	0.55	6	4.83	0.41	6	3.6%	-0.63 [-1.80, 0.54]
Warfarin(c)[29]	2.5	1.1	7	3.7	1.8	15	4.0%	-0.71 [-1.64, 0.21]
Warfarin(d)[29]	3.2	1.9	8	3.5	1.6	16	4.1%	-0.17 [-1.02, 0.68]
Warfarin(e)[29]	2.9	1.6	6	3.8	1.9	12	3.9%	-0.47 [-1.47, 0.52]
**Subtotal (95% CI)**			**31**			**62**	**19.1%**	**-0.55 [-0.99, -0.10]**
Heterogeneity: Tau² = 0.00; Chi² = 1.55, df = 4 (P = 0.82); I² = 0%								
Test for overall effect: Z = 2.39 (P = 0.02)								
**1.3.5 3K3A-APC**								
3K3A-APC[31]	2.94	1.03	5	1.01	0.49	5	2.7%	2.16 [0.42, 3.90]
3K3A-APC(b)[31]	18.49	4.52	5	7.53	2.8	5	2.4%	2.63 [0.70, 4.57]
3K3A-APC(c)[31]	9.29	2.43	10	4.24	1.87	10	3.6%	2.23 [1.06, 3.40]
3K3A-APC(d)[32]	6.05	1.1	5	1.77	1.63	5	2.3%	2.78 [0.78, 4.78]
3K3A-APC(e)[32]	9.32	1.49	8	3.68	0.74	8	2.3%	4.53 [2.48, 6.59]
**Subtotal (95% CI)**			**33**			**33**	**13.3%**	**2.65 [1.91, 3.40]**
Heterogeneity: Tau² = 0.01; Chi² = 4.03, df = 4 (P = 0.40); I² = 1%								
Test for overall effect: Z = 6.98 (P < 0.00001)								
**1.3.6 Others**								
APC(36-39)[14]	11.54	1.87	8	7.91	4.9	14	4.0%	0.85 [-0.06, 1.76]
Apixaban[24]	4.49	2.78	4	3.62	2.09	10	3.6%	0.36 [-0.81, 1.53]
BAY 60-6583[26]	3.82	0.95	17	2.53	1.01	17	4.3%	1.28 [0.54, 2.03]
JAQ1[30]	-4.67	1.41	9	-6.33	0.99	9	3.8%	1.30 [0.26, 2.34]
rADAMTS13[33]	2.9	0.35	8	1.32	0.5	8	2.7%	3.46 [1.77, 5.16]
Revacept[17]	2.33	1.29	9	2.14	1.29	9	4.0%	0.14 [-0.79, 1.07]
**Subtotal (95% CI)**			**55**			**67**	**22.5%**	**1.08 [0.39, 1.77]**
Heterogeneity: Tau² = 0.45; Chi² = 13.56, df = 5 (P = 0.02); I² = 63%								
Test for overall effect: Z = 3.07 (P = 0.002)								
**Total (95% CI)**			**200**			**275**	**100.0%**	**0.91 [0.49, 1.32]**
Heterogeneity: Tau² = 0.91; Chi² = 104.61, df = 28 (P < 0.00001); I² = 73%								
Test for overall effect: Z = 4.25 (P < 0.0001)								
Test for subgroup differences: Chi² = 76.01, df = 5 (P < 0.00001), I² = 93.4%								

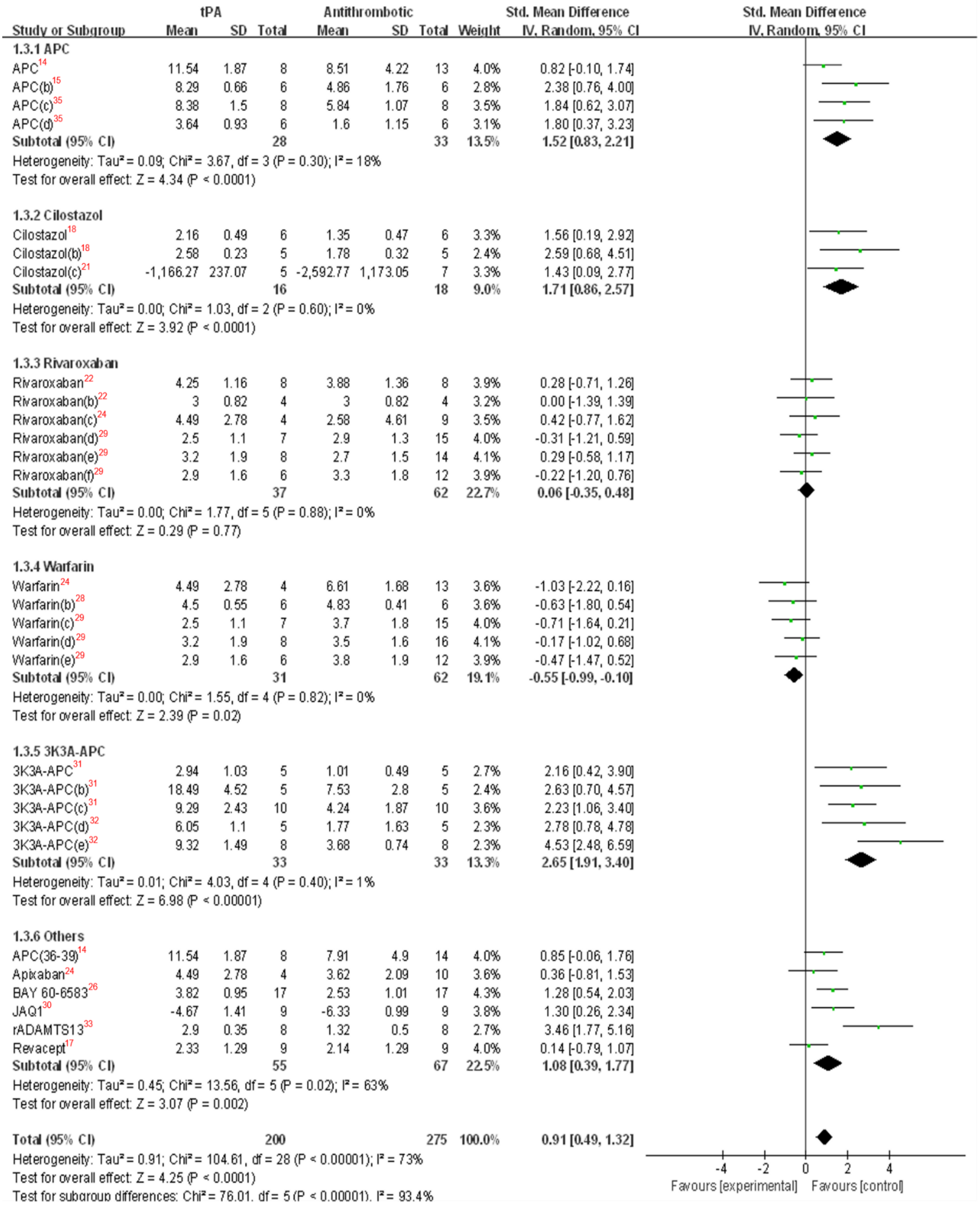

The horizontal lines indicate 95% confidence intervals for individual standardized mean differences (SMD), and the black diamonds indicates global SMD and confidence interval.

Figure S4. Effect of blinded assessment on the improvement in cerebral hemorrhage (A), infarct volume (B), and neurobehavioral score (C). T

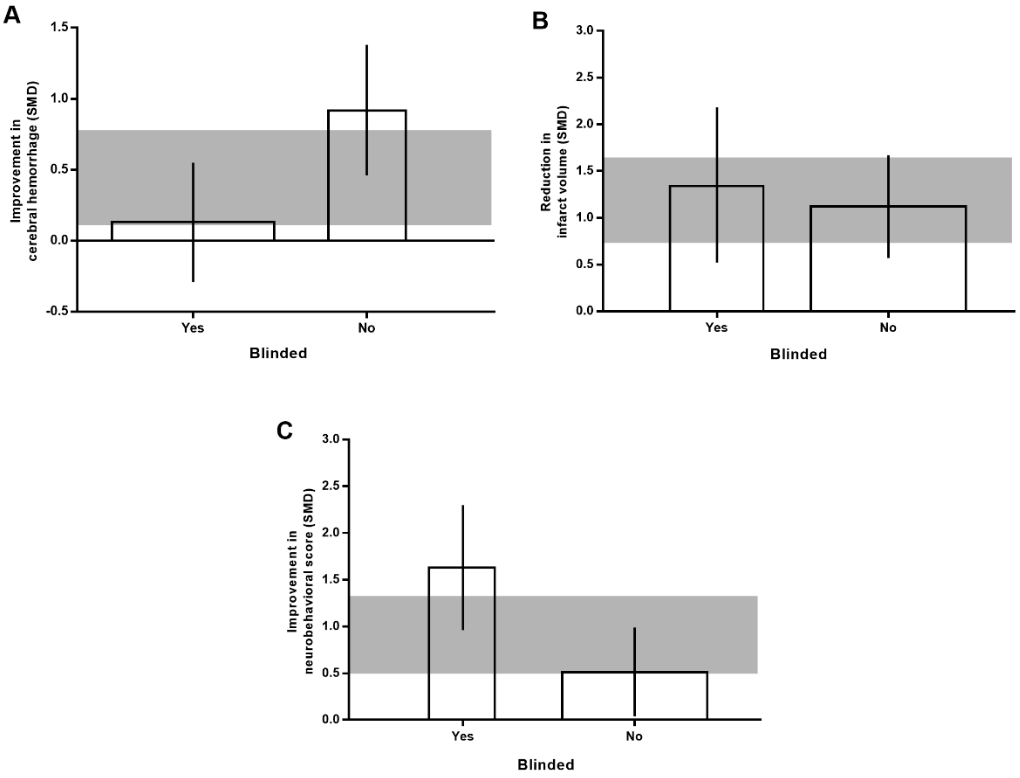

The width of each bar represent the relative number of animals in that subgroup; vertical error bars represent the 95% confidence interval for the individual estimates; and the horizontal grey bars represent the 95% confidence interval of the pooled estimate of efficacy.

Figure S5. Effect of sample size estimate on the improvement in cerebral hemorrhage (A), infarct volume (B), and neurobehavioral score (C).

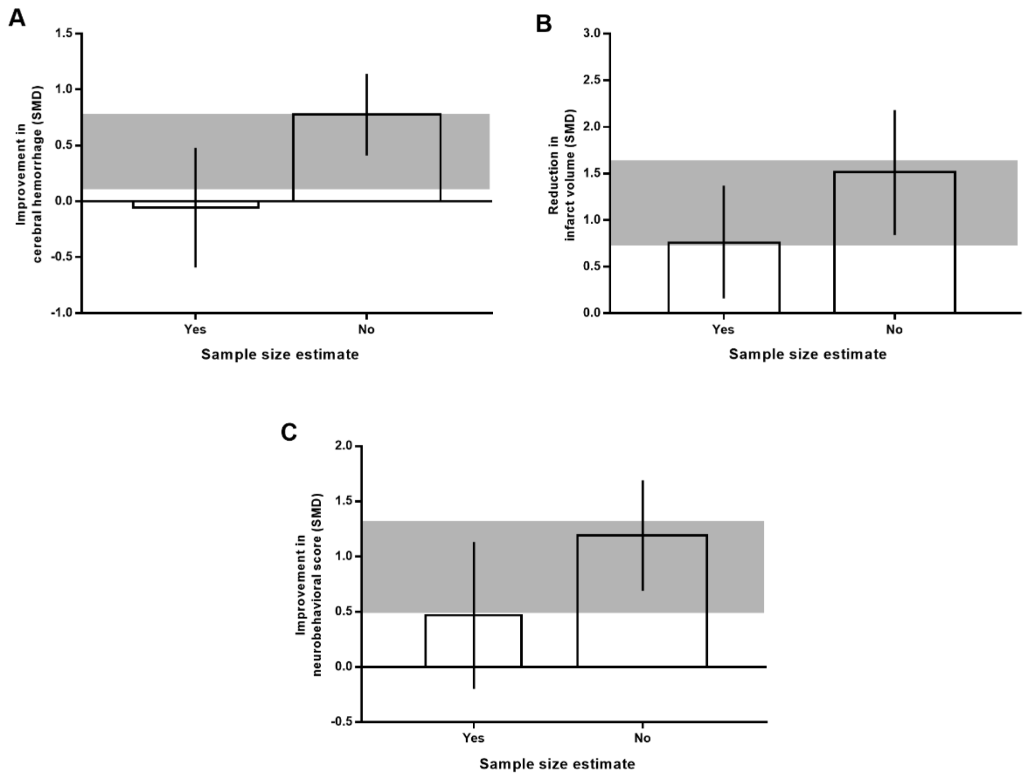

The width of each bar represent the relative number of animals in that subgroup; vertical error bars represent the 95% confidence interval for the individual estimates; and the horizontal grey bars represent the 95% confidence interval of the pooled estimate of efficacy.

Figure S6. Effect of stroke model used on the improvement in cerebral hemorrhage (A), infarct volume (B), and neurobehavioral score (C).

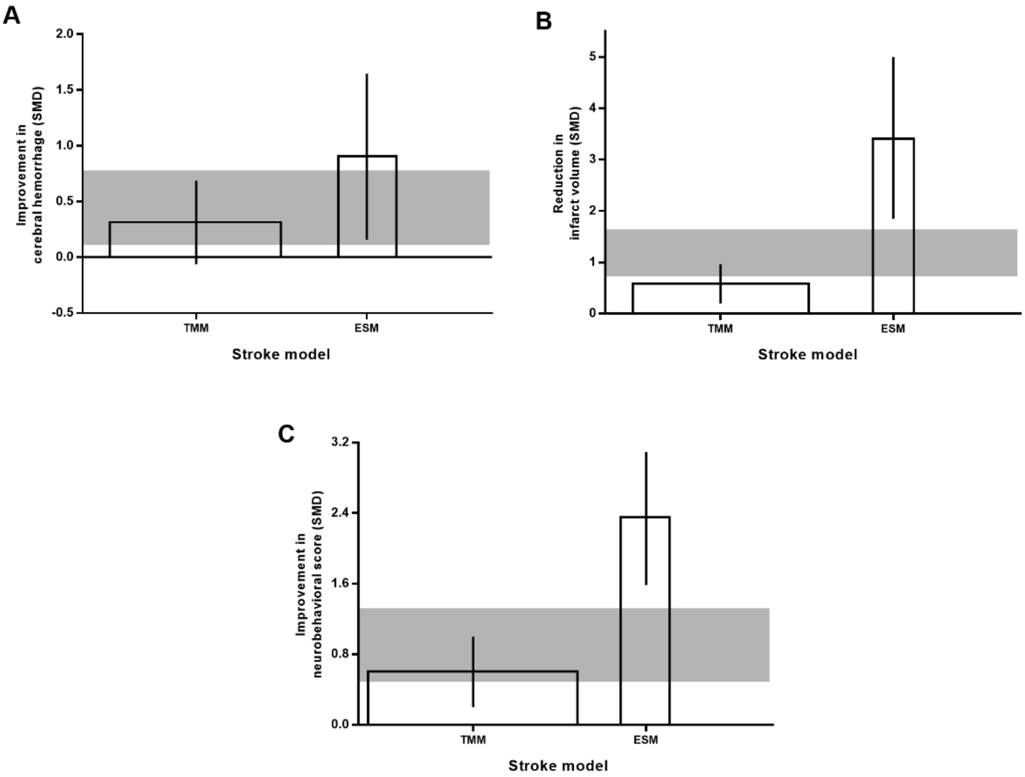

The width of each bar represent the relative number of animals in that subgroup; vertical error bars represent the 95% confidence interval for the individual estimates; and the horizontal grey bars represent the 95% confidence interval of the pooled estimate of efficacy.

Figure S7. Effect of animal species used on the improvement in cerebral hemorrhage (A), infarct volume (B), and neurobehavioral score (C).

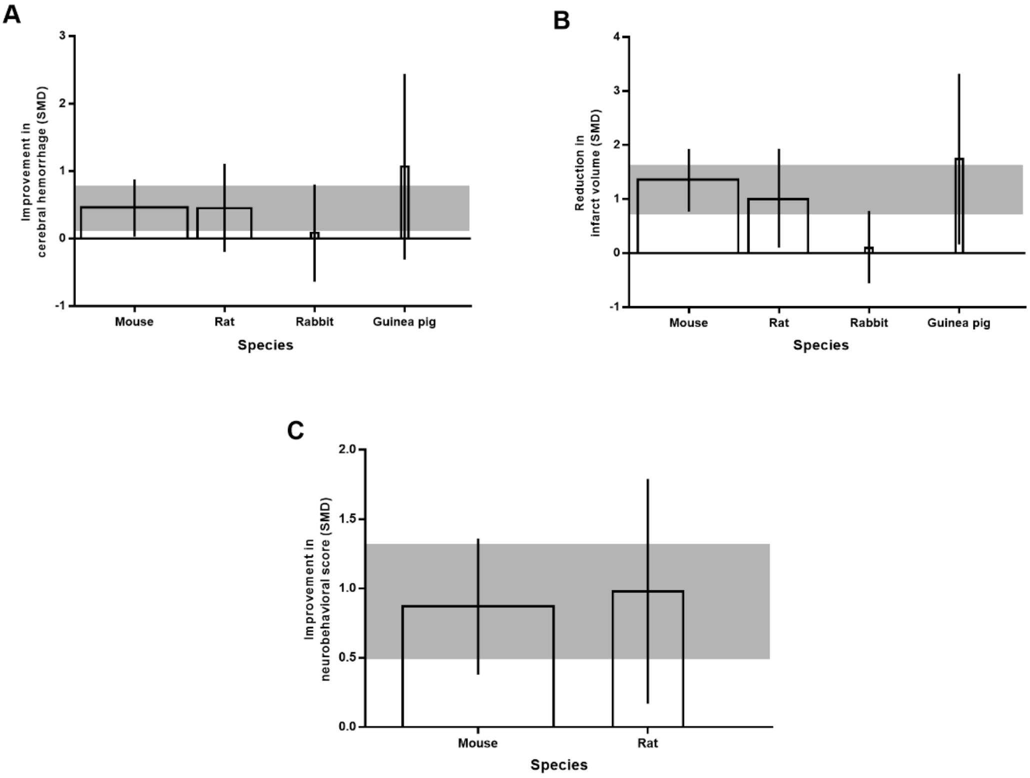

The width of each bar represent the relative number of animals in that subgroup; vertical error bars represent the 95% confidence interval for the individual estimates; and the horizontal grey bars represent the 95% confidence interval of the pooled estimate of efficacy.

Figure S8. Effect of drug administration time on the improvement in cerebral hemorrhage (A), infarct volume (B), and neurobehavioral score (C).

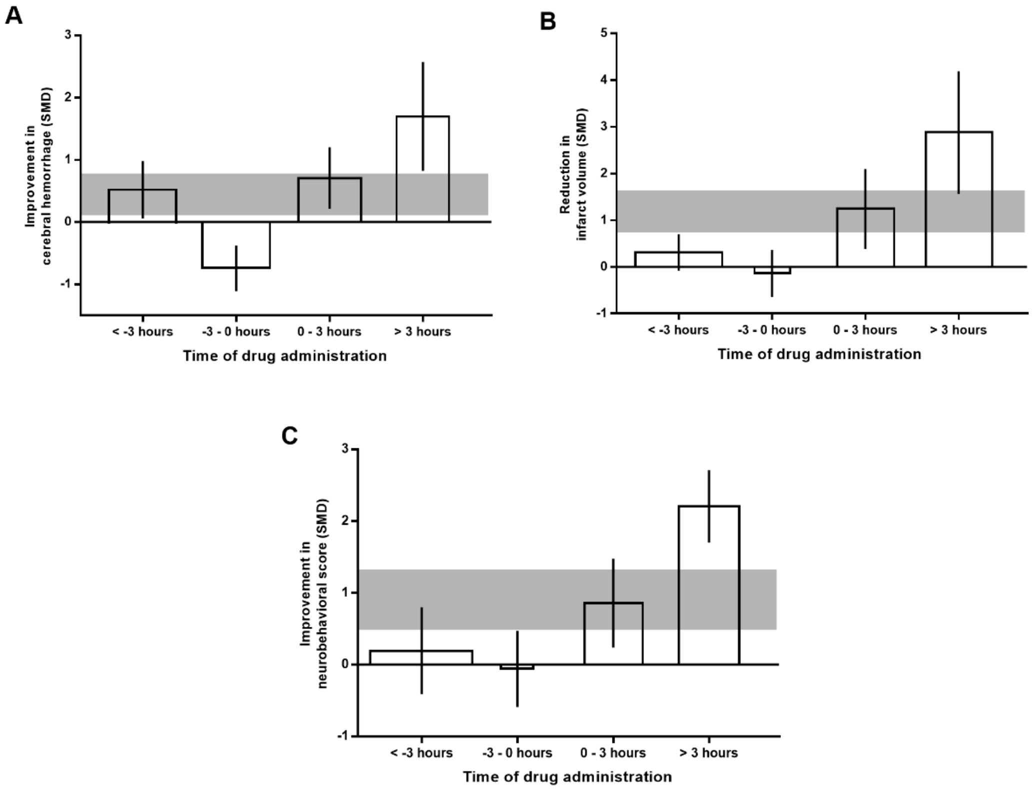

The width of each bar represent the relative number of animals in that subgroup; vertical error bars represent the 95% confidence interval for the individual estimates; and the horizontal grey bars represent the 95% confidence interval of the pooled estimate of efficacy.

Figure S9. Funnel plots for cerebral hemorrhage (A), infarct volume (B), and neurobehavioral score (C) showing the publication bias.

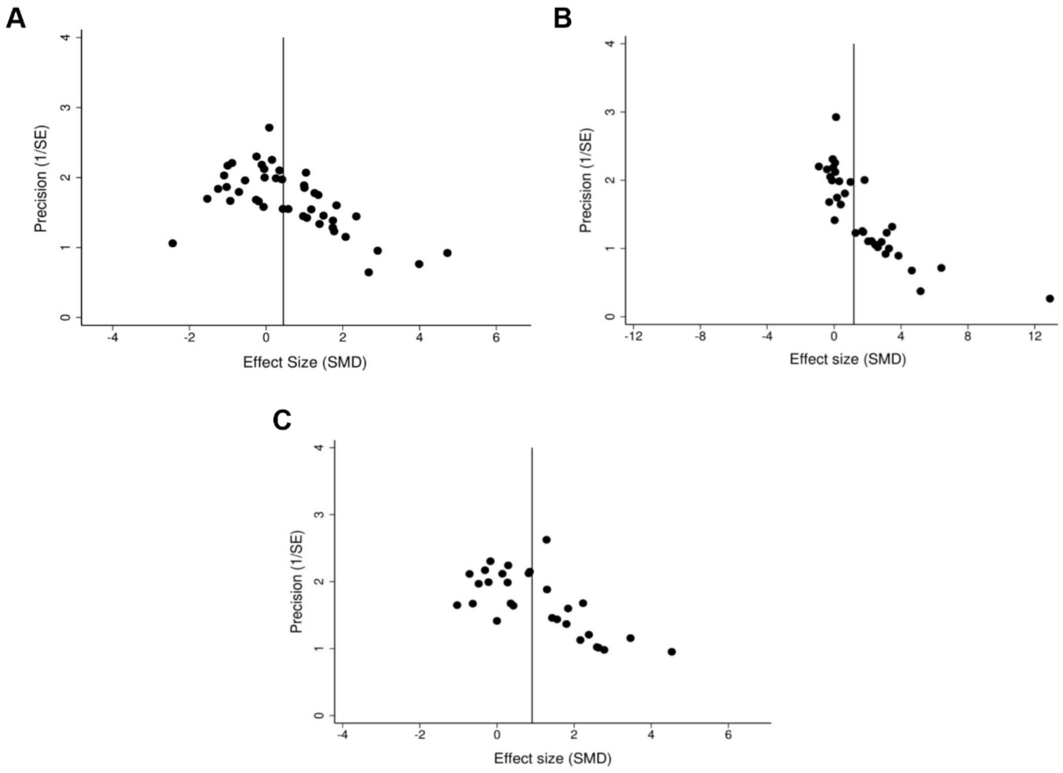

The vertical lines represent the pooled estimate of efficacy. The black circles represent the published studies.

Figure S10. Sensitivity analysis for cerebral hemorrhage (A), infarct volume (B), and neurobehavioral score (C) evaluating the robustness of the results.

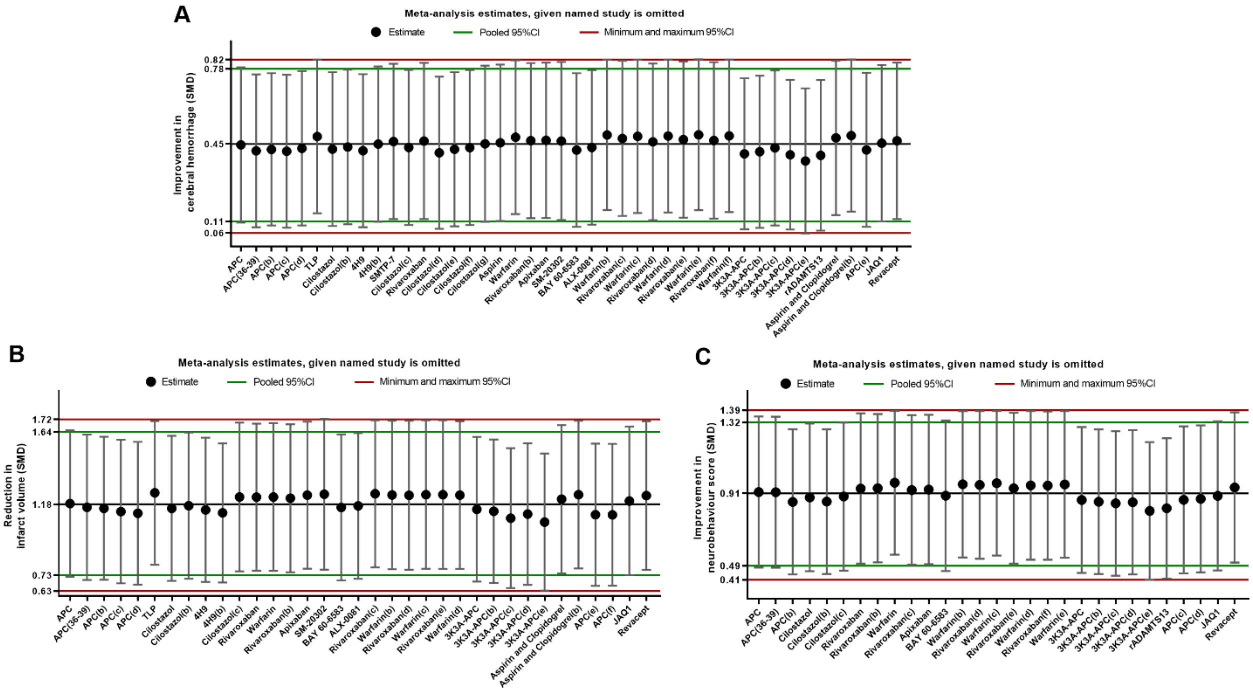

The vertical error bars represent the 95% confidence interval for the individual estimates. The horizontal red bars represent the potentially minimum and maximum 95% confidence interval and the horizontal green bars represent the actual 95% confidence interval of the pooled estimate of efficacy.
